# Construction and validation of a prognostic model for kidney renal clear cell carcinoma based on podocyte‐associated genes

**DOI:** 10.1002/cam4.4733

**Published:** 2022-04-04

**Authors:** Can Chen, Rui‐Xia Yang, Jie‐Xin Zhang, Hua‐Guo Xu

**Affiliations:** ^1^ Department of Laboratory Medicine the First Affiliated Hospital of Nanjing Medical University Nanjing China; ^2^ Branch of National Clinical Research Center for Laboratory Medicine Nanjing China

**Keywords:** prognostic model, KIRC, podocyte‐associated gene, overall survival, immune cell infiltration

## Abstract

**Background:**

As the most common renal malignancy, kidney renal clear cell carcinoma (KIRC) has a high prevalence and death rate as well as a poor response to treatment. Developing an efficient prognostic model is essential for accurately predicting the outcome and therapeutic benefit of KIRC patients.

**Methods:**

Gene expression profiles of podocyte‐associated genes (PAGs) were obtained from The Cancer Genome Atlas and GEO datasets. Cox regression and Lasso regression analyses were then used for filtering prognosis‐associated PAGs. Risk score (RS) was computed from these genetic characteristics. Kaplan–Meier analysis and receiver operating characteristic (ROC) curves were applied for ascertaining the prognostic value. Stratified analysis was used to sufficiently validate model performance. Concordance index was used to compare the predictive ability of different models. Immuno‐infiltration analysis and immunophenoscore were utilized for the prediction of patient reaction to immune checkpoint inhibitors (ICIs).

**Results:**

WT1, ANLN, CUBN, OSGEP, and RHOA were significantly associated with KIRC prognosis. Prognostic analysis indicated that high‐RS patients have a significantly poorer outcome. Cox regression analysis demonstrated a potential for RS to be an independent prognostic factor. Pathway enrichment results indicated a lower enrichment of cancer‐related biological pathways in the low‐RS subgroup. Immune infiltration analysis and IPS demonstrated greater responsiveness to ICIs in the high RS group.

**Conclusions:**

This podocyte‐associated KIRC prognostic model can effectively predict KIRC prognosis and immunotherapy response, which may help to provide clinicians with more effective treatment strategies.

## Graphical abstract

We constructed a prognostic model of podocyte‐associated genes in KIRC, which is an accurate and reliable marker for predicting prognosis in KIRC and may provide new ideas for clinical treatment.

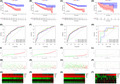

## 1 INTRODUCTION

Kidney renal clear cell carcinoma (KIRC) is the commonest pathological subtype of human urinary system tumors, accounting for about 85–90%.^1^ In the early stage of KIRC, only a few patients have obvious symptoms, such as hematuria, abdominal masses.^2^ The 5‐year survival rate of early KIRC patients after surgical treatment can reach 80–90%.^3^ However, patients with advanced KIRC are usually less sensitive to treatment and have a poor prognosis accompanied by distant metastases. It is estimated that there might be approximately 13,780 deaths from KIRC in the US in 2021.^4^ With rapid advances in molecular biology and bioinformatics, several studies have identified many genes relevant to the KIRC progression that can be used as potential prognostic biomarkers.^5^, ^6^ However, for the time being, a reliable marker for detecting early KIRC is still lacking, and studies have demonstrated that high tumor heterogeneity and delayed detection are major factors contributing to untimely treatment and recurrence.^7^ Therefore, determining efficient biological markers for early identification and prediction of KIRC survival and establishing prognostic‐related models have extremely important clinical significance for the treatment and prognosis of patients.^8^ By establishing a prognostic model, clinicians can make better individualized survival predictions at the molecular level.

Podocytes, also known as epithelial cells of the visceral layer of the renal capsule, are attached to the outer glomerular basement membrane and are a major component of glomerular filtration barrier.^9^, ^10^ Lesions of the podocytes have been found to cause podocytopathies, including minimal change nephrosis and focal segmental glomerulosclerosis, which are typically clinically manifested as persistent proteinuria.^11^, ^12^, ^13^ A large number of podocyte‐associated proteins and their coding genes have been identified through molecular biology sequencing, and mutations or loss of these genes can lead to the development and exacerbation of steroid‐resistant nephrotic syndrome.^14^, ^15^

Currently, there are few researches on podocytes and their related genes and KIRC. We sought to investigate the clinical value of podocytes in KIRC through the integration of podocyte‐associated gene (PAG) expression profiles with clinical data from KIRC patients. First, we extracted the expression levels of more than 50 PAGs from The Cancer Genome Atlas (TCGA) database and randomly divided the clinical patients into a training and internal validation cohort. In training cohort, LASSO regression and multivariate Cox regression were performed to define PAGs significantly related to the prognosis of KIRC patients and to establish a podocyte‐related prognosis model. Second, Kaplan–Meier (K–M) curves and receiver operating characteristic (ROC) curves were drawn in the training, internal validation, TCGA total validation, and Gene Expression Omnibus (GEO) external validation cohorts to ascertain the model's reliability. In addition, we evaluated the effectiveness of this prognostic model in predicting patients to receive immunotherapy. In summary, the podocyte‐associated risk model can serve an essential function in individualized and precise treatment for KIRC patients.

## 2 METHODS AND MATERIALS

### 2.1 KIRC data acquisition and preprocessing

The TCGA and GEO databases were searchable for podocyte‐associated gene expression profiles and full clinical annotations. Two eligible KIRC cohorts (GSE29609 and TCGA‐KIRC) were collected for further analysis.^16^ Patients lacking complete clinical information and duplicate samples were excluded for further evaluation. Finally, 518 samples from TCGA database and 39 samples from GSE29609 dataset were subject to further analysis. The samples in the TCGA database (*n* = 518) were further randomly allocated to the training and internal validation cohort in a 1:1 ratio. For the dataset in TCGA, RNA sequencing data for gene expression (FPKM values) were obtained directly from UCSC Xena platform (https://xena.ucsc.edu/) and then converted to transcript per kilobase million (TPM) values. The normalized expression matrix for the microarray data in the GSE29609 dataset was downloaded via the GEO website (https://www.ncbi.nlm.nih.gov/geo/). The "sva" package eliminated the batching effect.^17^ Clinical information on KIRC patients is concluded in Table S1.

### 2.2 Gene ontology and Kyoto Encyclopedia of Genes and Genomes pathway analysis

"ClusterProfiler" package was available for^18^ gene ontology (GO)^19^ and Kyoto Encyclopedia of Genes and Genomes (KEGG) pathways analysis.^20^ Adjusted *p* values <0.05 for pathways were deemed statistically significant.

### 2.3 Construction of prognostic model

The training cohort was employed for creating the prognostic model and internal validation cohort, TCGA total cohort, and GSE29609 cohort were utilized to assess the accuracy of the model. Based on previous studies of PAGs, we identified 53 genes for inclusion in this study.^14^ First, we used univariate Cox regression on 53 PAGs in the training cohort to further screen out PAGs related to KIRC prognosis. Among them, when the HR was less than 1 and *p* < 0.05, the gene was considered a protective factor. Conversely, it was a risk gene when HR was greater than 1 and *p* > 0.05. Then, least absolute shrinkage and selection operator (Lasso) regression analysis could be applied to eliminate highly relevant survival‐related PAGs and avoid overfitting of the model.^21^, ^22^ Finally, five PAGs were determined by multivariate Cox regression (Table 1). On this basis, we calculated risk scores (RS) for individuals with KIRC in each of the four cohorts, including the training set, internal validation cohort, TCGA total cohort, and GSE29609 cohort. RS was calculated as follows: RS = $\sum_{i=1}^{n}\text{Coefi}*\text{Expi}$. ("Coefi" is the correlation coefficient of the gene in the multivariate Cox analysis, and "Expi" is the level of gene expression.)

**TABLE 1:** Univariate and multivariate Cox regression analyses of the survival‐related PAGs of KIRC

id	Univariate analysis		Multivariate analysis		
	HR (95% CI)	*p*‐value	coef	HR (95% CI)	*p*‐value
ARHGAP24	0.509 (0.420–0.615)	<0.001			
ANLN	1.743 (1.468–2.070)	<0.001	0.271	1.312 (1.084–1.587)	0.005
CUBN	0.788 (0.731–0.849)	<0.001	−0.210	0.811 (0.745–0.882)	<0.001
LAGE3	1.791 (1.449–2.213)	<0.001			
RHOA	0.421 (0.298–0.597)	<0.001	−0.565	0.568 (0.403–0.801)	0.001
NUP85	2.287 (1.610–3.249)	<0.001			
LAMB2	0.652 (0.543–0.784)	<0.001			
CDC42	0.412 (0.279–0.608)	<0.001			
PDSS2	0.465 (0.331–0.653)	<0.001			
WT1	1.392 (1.199–1.616)	<0.001	0.262	1.300 (1.091–1.549)	0.003
OSGEP	2.175 (1.524–3.104)	<0.001	0.562	1.755 (1.227–2.510)	0.002
NUP133	0.496 (0.356–0.690)	<0.001			
DLC1	0.682 (0.564–0.826)	<0.001			
CD2AP	0.614 (0.479–0.788)	<0.001			
ITSN2	0.566 (0.413–0.775)	<0.001			
DGKE	0.492 (0.327–0.742)	0.001			
NUP93	2.517 (1.436–4.411)	0.001			
TRPC6	0.630 (0.474–0.839)	0.002			
ACTN4	0.656 (0.504–0.852)	0.002			
AVIL	1.643 (1.206–2.238)	0.002			
SCARB2	0.595 (0.430–0.824)	0.002			
NUP107	2.143 (1.327–3.462)	0.002			
KANK1	0.697 (0.543–0.894)	0.005			
XPO5	1.623 (1.145–2.299)	0.006			
RAC1	1.836 (1.135–2.971)	0.013			
NUP205	0.690 (0.504–0.945)	0.021			
MYO1E	0.734 (0.560–0.963)	0.026			

Abbreviation: PAG, podocyte‐associated gene.

### 2.4 Performance verification of prognostic models

Based on the median RS, we divided patients into high and low‐risk groups. In four cohorts, we plotted K–M curves to compare the difference in overall survival (OS) between two groups, and plotted time‐dependent ROC curves to assess the predictive reliability of the model. Risk stratification analysis was conducted across the TCGA cohort to confirm the predictability of the risk model within each clinical cohort. The concordance index (C‐index) was used to compare the predictive performance between different models.

### 2.5 Construction of prognostic nomogram

Cox regression analysis was performed on clinicopathological parameters and RS from the entire TCGA cohort to confirm that RS might be an independent prognostic factor for patients with KIRC. Screened independent prognostic factors were integrated into the plotting of the nomogram to predict the patient's 1, 3, and 5‐year OS rate.

### 2.6 Gene Set Enrichment Analysis

The Gene Set Enrichment Analysis (GSEA) algorithm could assess whether a predefined set of genes are statistically significantly and consistently different in two biology states.^23^ GSEA software (version 4.0.3) was used to identify pathways that were enriched in two different risk groups. *p* < 0.05 and false discovery rate (FDR) < 0.25 were considered as cutoff values.

### 2.7 Immune cell infiltration analysis

According to the normalized PAGs expression profile, we quantified 22 infiltrating immune cells in the KIRC tumor immune microenvironment (TIME) using CIBERSORT, a deconvolution algorithm.^24^ The sum of all estimates of the immune cell ratio was equal to 1 for each sample. We then used the ssGSEA (Single Sample Gene Set Enrichment Analysis) algorithm to quantify the relative infiltration abundance of immune cells in KIRC TIME. Gene set for marking immune infiltrating cell types was derived from Charoentong's study.^25^, ^26^

### 2.8 IPS analysis

By analyzing gene expression in the four cell types, machine learning could be applied to impartially derive the patient's IPS.^25^ IPS was calculated according to gene expression of representative cell types, ranging from 0 to 10. The higher the IPS, the higher the immunogenicity.^25^ The patient's IPS was obtained from The Cancer Immunome Atlas (TCIA) (https://tcia.at/home).

### 2.9 Statistical analysis

All statistical analysis and graphics were done using R 4.0.3. Cox regression analysis was performed to identify PAGs associated with survival and to demonstrate the independence of prognostic indicators. Lasso regression analysis was employed to remove highly relevant genes and prevent overfitting of the model. The K–M curve displayed the OS difference between the two groups and the log‐rank test was applied to determine the significant nature of the difference. ROC curves and corresponding AUCs were applied to assess the accuracy of this model. Nomogram showed the prognosis of RS in KIRC patients more intuitively. P<0.05 was regarded as statistically different.

## 3 RESULTS

### 3.1 The genetic variation landscape of podocyte‐associated genes in KIRC

**FIGURE 1:**
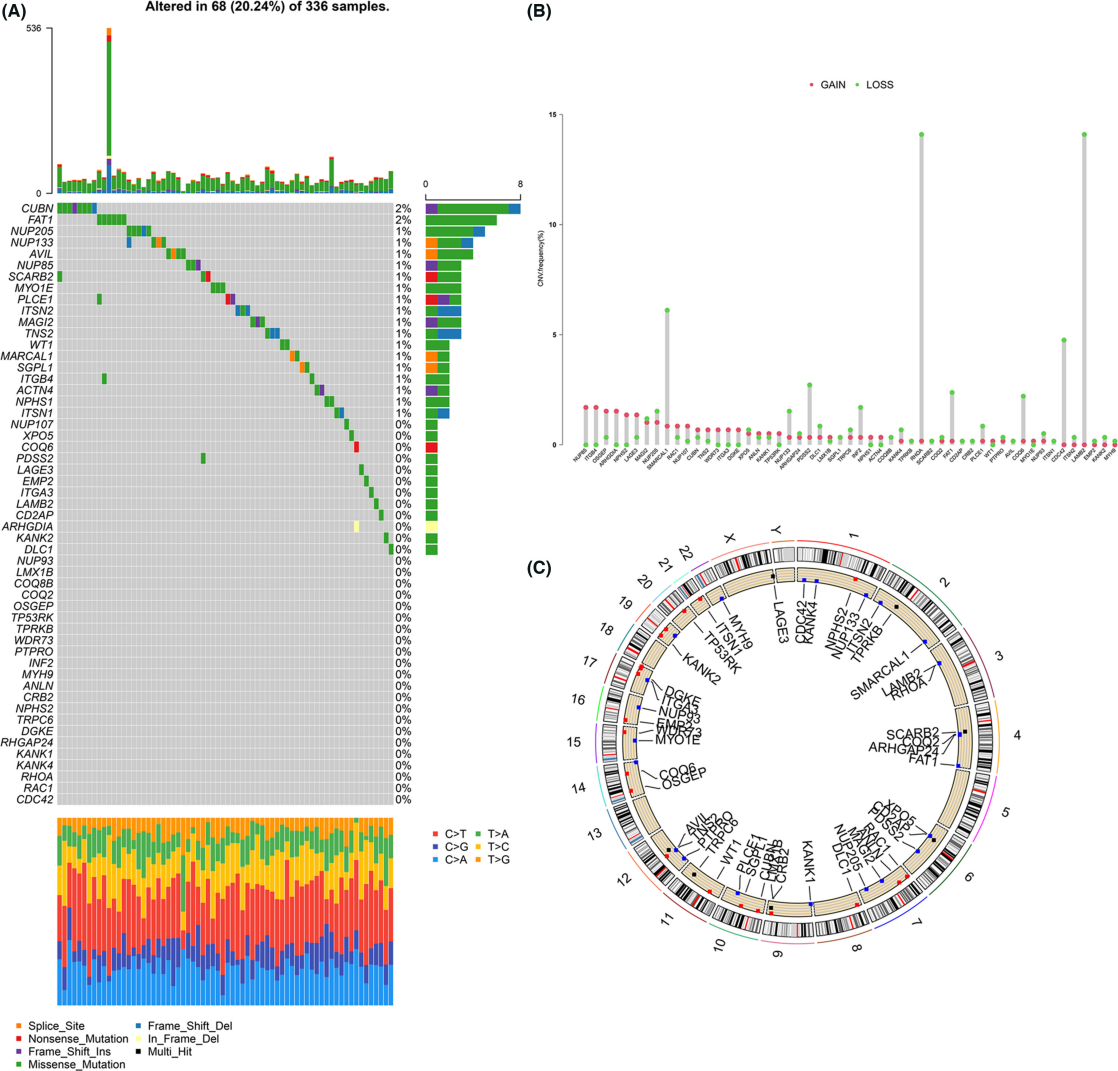
Landscape of genetic variation of podocyte‐associated genes (PAGs) in kidney renal clear cell carcinoma (KIRC). (A) The mutation frequency of 53 PAGs in 336 KIRC patients from The Cancer Genome Atlas‐KIRC cohort. (B) The CNV variation frequency of 53 PAGs. (C) The location of CNV alteration of PAGs on 23 chromosomes

53 PAGs were finally identified. We summarized the incidence of copy number variation (CNV) in the 53 PAGs in KIRC. Mutations in PAGs occurred in 68 out of 336 samples, with a frequency of 20.24%. CUBN and FAT1 were found to exhibit the highest mutation frequencies (Figure 1A). Subsequently, the investigation of the frequency of CNV alterations showed that CNV alterations was prevalent in 53 PAGs, with genes showing copy number amplification or deletion in approximately equal proportions. Among them, SMARCAL1, RHOA, and LAMB2 showed extensive CNV deletion frequencies (Figure 1B). The location of CNV changes in PAGs on chromosomes was depicted in Figure 1C. In order to ascertain whether the above genetic variants affect PAGs expression in KIRC, we compared the mRNA expression levels of PAGs between normal and KIRC samples and observed that altered CNV may be responsible for the altered expression of PAGs. PAGs with deleterious CNVs were less expressed in KIRC than in normal tissues (such as RHOA and PDSS2) and vice versa (such as NUP85 and ITGB4) (Figure 1B; Table S1). These analyses indicated that there was considerable heterogeneity in the genetic and expression alterations of PAGs between normal and KIRC samples, implying that imbalances in PAG expression were critical in the development and progression of KIRC.

### 3.2 Pathways and biological processes involving podocyte‐associated genes

**FIGURE 2:**
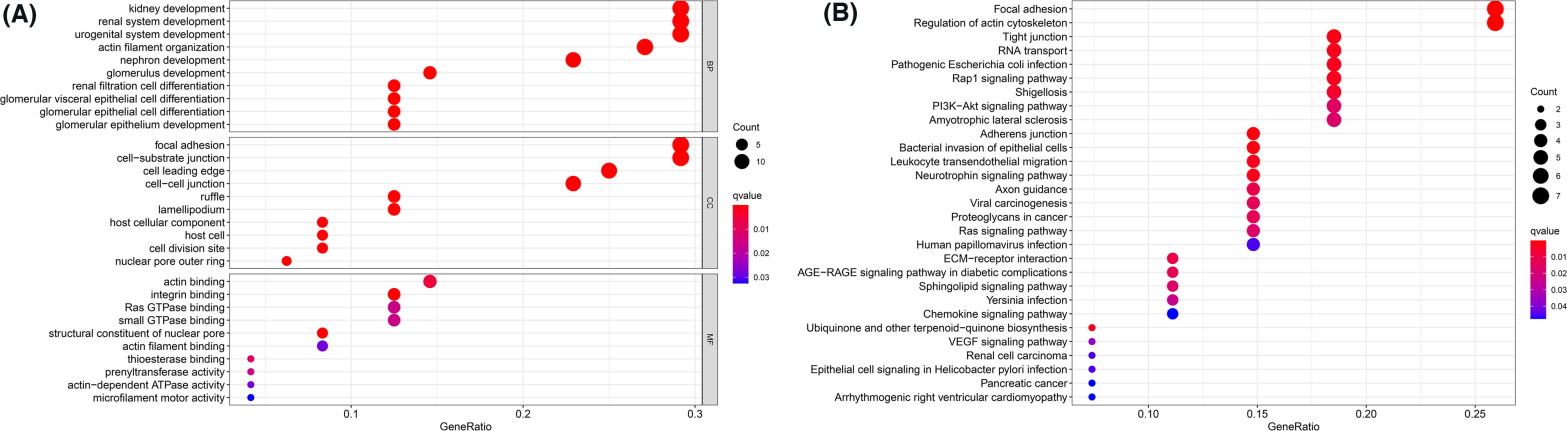
Functional and pathway enrichment analyses of 53 podocyte‐associated genes (PAGs). (A) Bubble plot representing the enriched GO terms of PAGs. (B) Bubble plot representing the enriched Kyoto Encyclopedia of Genes and Genomes pathways of PAGs

To assess the mechanisms of PAGs, GO, and KEGG pathway enrichment analysis were performed (Table S2). Figure 2 summarized the significant enrichment of biological processes. Clearly, these genes showed biological processes significantly associated with kidney growth and cell migration, including kidney development, focal adhesion, tight junction, renal cell carcinoma, etc. This confirmed that PAGs contributed significantly to the development of KIRC.

### 3.3 Developing a podocyte prognostic model on a training cohort

**FIGURE 3:**
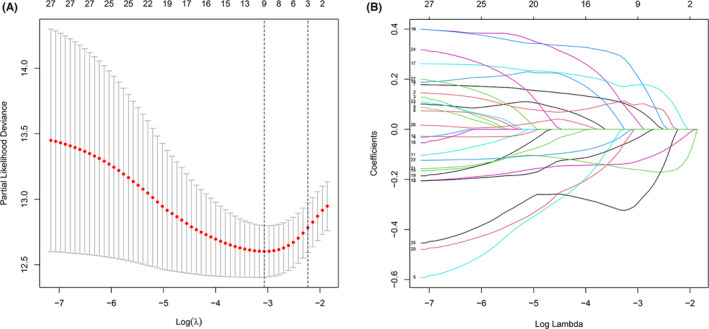
Screening of the optimal podocyte‐associated genes (PAGs) used for final construction of risk model using Lasso regression. (A) The optimal parameter was screened by cross validation. (B) LASSO coefficients profiles of 27 survival associated PAGs

We obtained relevant clinical information and PAGs expressions of KIRC patients from TCGA and GEO databases, and randomly divided the 518 specimens with survival information in the TCGA database into training and internal validation cohorts. In training cohort, we ran univariate Cox regression analyses on 53 PAGs and then screened 27 PAGs for significant association with KIRC survival (*p* < 0.05, Table 1). Subsequently, we applied Lasso regression analysis to remove highly relevant PAGs (Figure 3). Lastly, five PAGs were obtained from multivariate Cox regression analysis (WT1, OSGEP, ANLN, CUBN, and RHOA) (Table 1). According to the correlation coefficients, we calculated RS and established a risk model. RS = (0.26237*WT1) + (0.56227*OSGEP) + (−0.20967*CUBN) + (0.27124*ANLN) + (−0.56505* RHOA).

### 3.4 Performance verification of prognostic model

**FIGURE 4:**
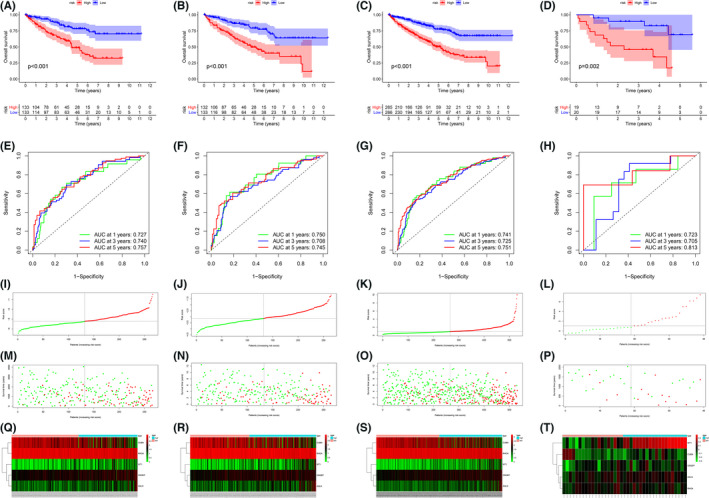
Risk stratification analysis of the training cohort, internal validation cohort, total The Cancer Genome Atlas (TCGA) cohort and GSE29609 cohort. (A–D) Kaplan–Meier survival analysis of OS in high‐risk (red) and low‐risk(blue) kidney renal clear cell carcinoma (KIRC) patients in training (A), internal validation (B), total TCGA (C), and GSE29609 cohort (d). E‐H. ROC curves for predicting 1‐, 3‐, 5‐year OS in the training (E), internal validation (F), total TCGA (G), and GSE29609 cohort (H). (I–L) Risk score distribution of KIRC patients in training (I), internal validation (J), total TCGA (K), and GSE29609 cohort (L). (M–P) Scatterplots of KIRC patients with different OS status in training (M), internal validation (N), total TCGA (O), and GSE29609 cohort (P). (Q–T) Expression of five podocyte‐associated genes in KIRC patients with different risk status in training (Q), internal validation (R), total TCGA (S), and GSE29609 cohort (T)

To test the reliability of the risk model, K–M survival curves were created to assess the OS differences in each of the four cohorts. Using the median RS of the four individual queues as the respective threshold values, we divided patients into high and low‐risk groups by ranking RS from highest to lowest. As seen in Figure 4A–D, significant differences in OS were observed between high‐ and low‐risk groups in all four independent datasets, with patients in high group having lower survival rates than low‐risk group (*p* < 0.05). Time‐dependent ROC curves were also applied to validate the precision of this model in predicting survival at 1, 3, and 5 years for KIRC patients, with the corresponding AUC greater than 0.7 (Figure 4E–H). Moreover, we analyzed the distribution of RS and patients' survival status, with Figure 4I,M showing that the higher the RS of the patient, the higher the mortality rate. Heat map displayed the expression of PAGs in KIRC patients in different risk groups (Figure 4Q). These results could be fully validated in the internal validation cohort, total TCGA cohort and GSE29609 cohort (Figure 4I–T).

### 3.5 Correlation between risk score and clinical characteristics

**FIGURE 5:**
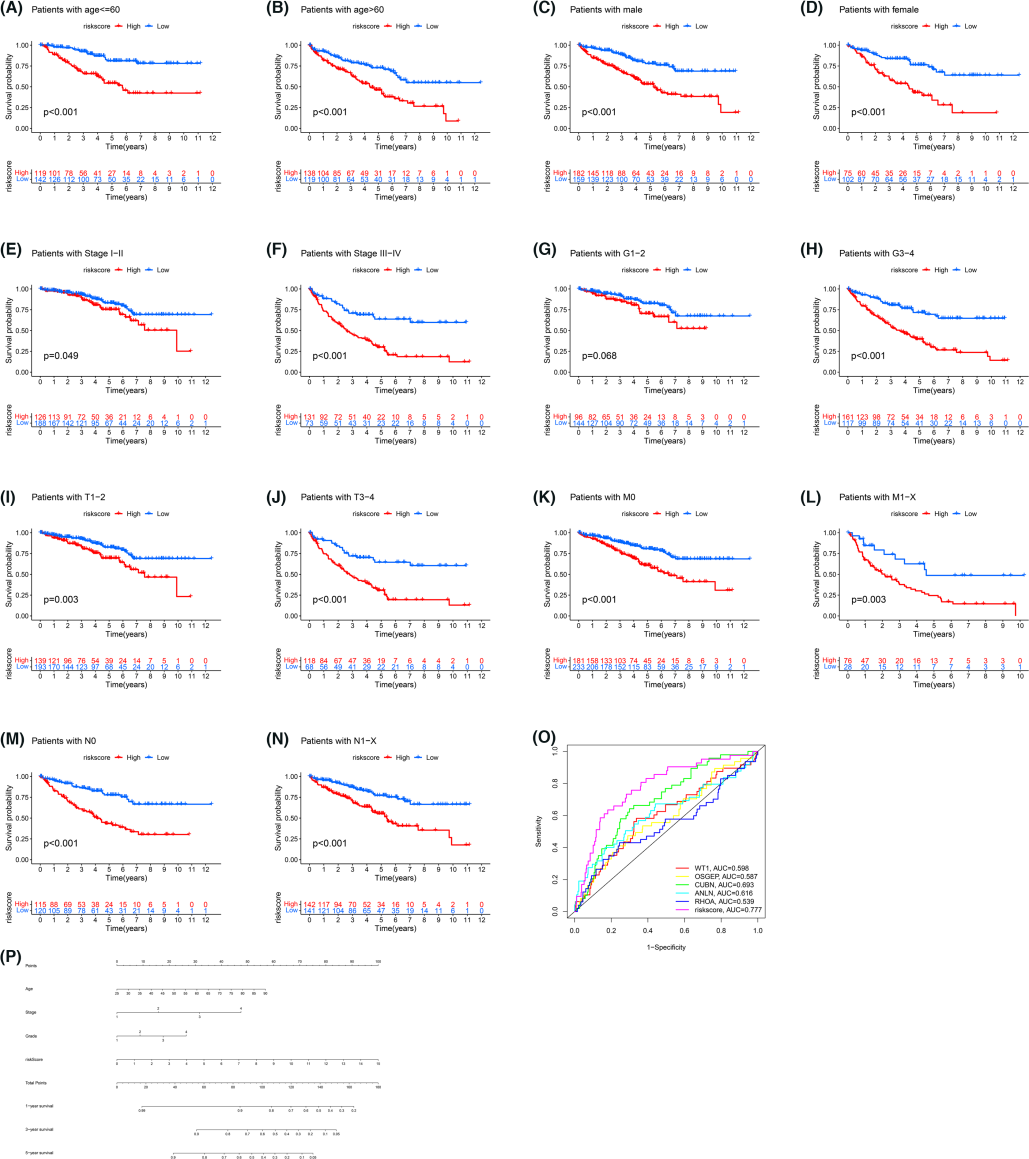
Performance of risk scores in different clinical stratifications. (A–M) Kaplan–Meier survival curves for RS and OS in different kidney renal clear cell carcinoma sub‐cohorts of patients, including male (A), female (B), ≤60 (C), >60 (D), stage1–2 (E), stage3–4 (F), grade1‐3 (G), grade3‐4 (H), T1‐2 (I), T3‐4 (J), M0 (K), M1‐x (L), N0 (M), N1‐x (N). (O). ROC curves comparing the prognostic performance of RS with five genes. (P). Nomograms incorporating RS and clinical characteristics for predicting patient OS

After removing those samples with missing clinical and survival information, we counted KIRC patient clinical data in four separate datasets to facilitate further investigation of the association of risk models with the clinical features of KIRC patients (Table S3). Considering the effect of sample size on statistical errors, we finally selected 518 specimens from the total TCGA cohort for the analysis. Based on different clinical characteristics, we divided all KIRC patients into several different subgroups, including age ≤ 60 (*n* = 261), age > 60 (*n* = 257), female (*n* = 177), male (*n* = 341), stage I–II subgroup (*n* = 314), stage III–IV (*n* = 204), grade I–II (*n* = 240), grade III–IV (*n* = 278), T I–II ( *n* = 332), T III–IV (*n* = 186), M0 (*n* = 414), M1‐X (*n* = 104), N0 (*n* = 235), and M1‐X (*n* = 283). In total TCGA database, survival rates were statistically different across clinical characteristics (including gender, stage, grading, and TMN staging) in both groups (*p* < 0.05, Figure 5A–N). Moreover, logistic analysis was carried out to investigate the association of RS with clinical features. The higher the risk value, the higher the tumor stage, and the more likely it was to metastasize (Table 2). Besides, we also constructed ROC curves to compare the prognostic performance of RS with that of the five genes. As seen in Figure 5O, the AUC values for RS were significantly higher than the other five genes (AUC = 0.777). Together, these results suggested that this model performed well in KIRC stratification analysis, indirectly demonstrating the risk model's prognostic value.

**TABLE 2:** The correlation between RS and clinical characteristics of KIRC patients (logistic regression)

Clinical characteristics	Risk score			
	OR	OR.95L	OR.95H	*p*‐value
Age				
>60 versus ≤60	1.283	0.898	1.837	0.172
Gender				
Male versus female	1.588	1.104	2.292	0.013
Stage				
Stage 3‐4 versus Stage 1‐2	2.693	1.875	3.891	<0.001
Grade				
G 3–4 versus G 1–2	2.020	1.426	2.873	<0.001
T				
T3–4 versus T1–2	2.346	1.627	3.405	<0.001
M				
M1‐X versus M0	3.352	2.118	5.428	<0.001
N				
N1‐X versus N0	0.985	0.697	1.39	0.930

We performed COX regression analysis to determine that RS could be considered as an independent predictive factor. As seen in Table 3, RS could be considered as an independent prognostic factor (*p* < 0.001). The screened valid independent clinical prognostic factors (age, stage, and grade) were then plotted in a nomogram along with RS to more visually demonstrate the efficiency of the prognostic risk model in predicting OS for patients (Figure 5P).

**TABLE 3:** Univariate and multivariate cox regression analyses of RS and clinicopathological variables in predicting OS

Parameters	Univariate analysis				Multivariate analysis			
	HR	HR.95L	HR.95H	P	HR	HR.95L	HR.95H	P
Age	1.0293	1.0158	1.0429	<0.001	1.0315	1.0171	1.0461	<0.001
Gender	0.9395	0.6863	1.2862	0.6970				
Stage	1.9054	1.6676	2.1772	<0.001	2.1860	1.6707	2.8603	<0.001
Grade	2.2977	1.8714	2.8212	<0.001	1.4163	1.1222	1.7876	0.0034
T	1.9323	1.6374	2.2803	<0.001	0.7263	0.5337	0.9885	0.0420
M	1.5074	1.2673	1.7930	<0.001	0.8730	0.6491	1.1741	0.3691
N	0.9234	0.8342	1.0220	0.1237				
RiskScore	1.2523	1.1880	1.3202	<0.001	1.2642	1.1787	1.3559	<0.001

### 3.6 Podocyte‐associated gene signature as a novel predictive model in KIRC

To further evaluate the predictive performance of our model, we compared it with four other risk models, including a four gene,^27^ a nine gene,^28^ a five gene,^29^ and a three gene^30^ signature. To make them comparable, we also calculated risk scores for each model using multivariate Cox regression analysis and plotted ROCs to describe the prognostic performance of each, based on the corresponding genes included in the four models. The samples were then divided into two groups of high‐ and low‐risk based on median RS. The difference in prognosis between the high‐ and low‐risk groups was significant in all four models (*p* < 0.001, Figure S1A–D). However, as can be seen from the ROC curves, the AUCs of all four models were lower than our prognostic model (Figure S1E–I). In addition, we calculated the C‐index to evaluate the predictive ability between the models. It was clear that our model had the highest C‐index at 0.70 (Figure S1J). Thus, our model had the best performance in predicting prognosis compared to other models.

### 3.7 Multiple tumor‐associated pathways were diminished in low‐risk group

**FIGURE 6:**
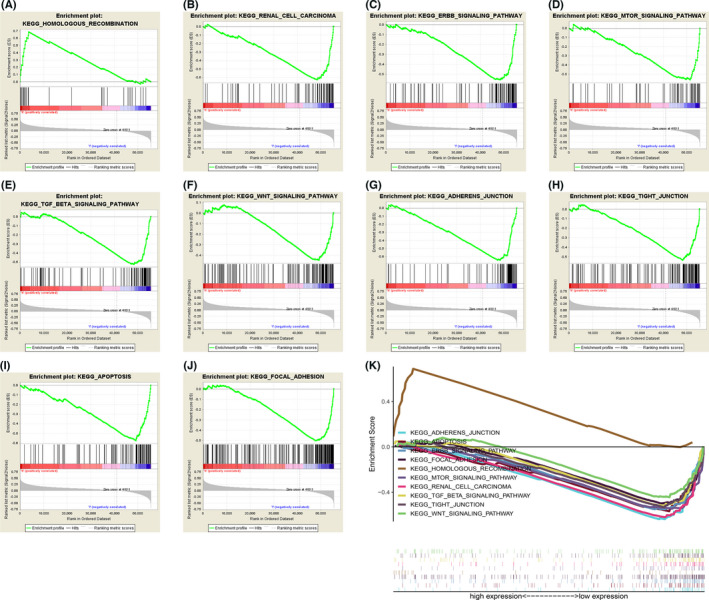
Five‐gene signature associated biological pathways in kidney renal clear cell carcinoma (KIRC). (A–J) Representative enriched pathways in high‐ and low‐risk KIRC patients through GSEA analysis. (A) Homologous recombination. (B) Renal carcinoma. (C) ERBB signaling pathway. (D) MTOR signaling pathway. (E) TGF‐BETA signaling pathway. (F) WNT signaling pathway. (g) Adherens junction. (H) Tight junction. (I) Apoptosis. (J) Focal adhesion. (K) GSEA of high RS patients and low‐RS patients

To thoroughly investigate the potential mechanisms and role of risk models in KIRC patient prognosis, we performed GSEA to screen for RS‐associated signaling pathways. GSEA results indicated that tumor‐related pathways such as homologous recombination were upregulated in high‐risk group (Figure 6A). In contrast, multiple pathways associated with KIRC development and metastasis, such as renal carcinoma, ERBB signaling pathway, MTOR signaling pathway, adherens junctions, tight junction, apoptosis, and focal adhesion were downregulated in low‐risk group (Figure 6B–K).

### 3.8 Differential immune infiltration and immunotherapy response in high‐ and low‐risk groups

**FIGURE 7:**
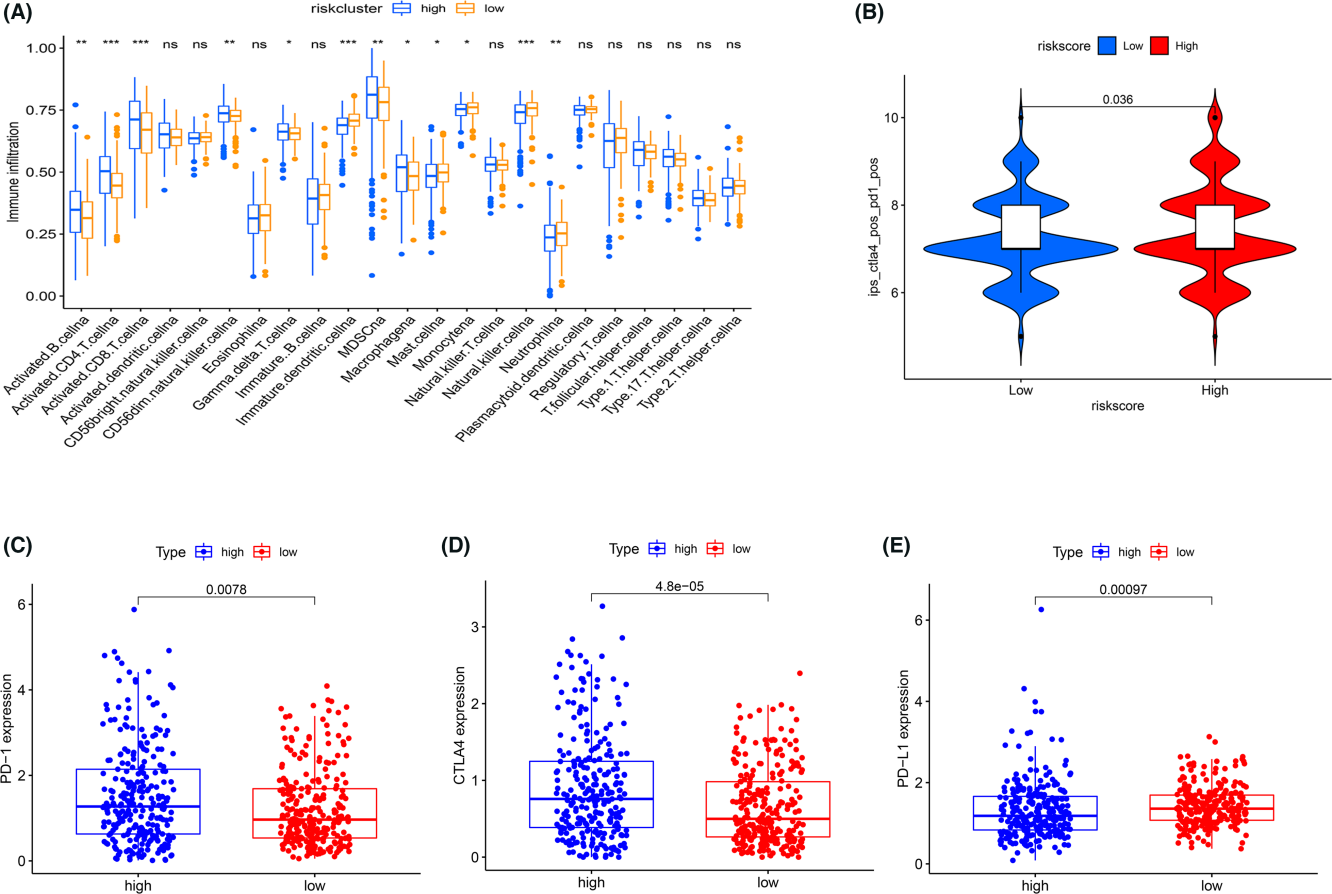
Tumor immune microenvironment (TIME) cell infiltration characteristics, immunophenoscore (IPS) and immunotherapy gene expression analysis in distinct risk groups. (A) The abundance of each kidney renal clear cell carcinoma TIME immune cell infiltration in high‐ and low‐risk group. (B) Differences in IPS (ips_ctla4_pos_pd1_pos) among low‐ and high‐risk groups. (C–E) Gene expression of PD‐1 (C), CTLA‐4 (D), and PD‐L1 (E) in low‐ and high‐risk groups. *p < 0.05; **p < 0.01; ***p < 0.001

GO and KEGG enrichment analyses yielded immune activation‐related pathways, including the chemokine signaling pathway, T cell costimulation, and positive regulation of T cell activation (Table S2), suggesting that PAGs were associated with TIME. To investigate the link between podocyte characteristics and immune infiltration, we performed correlation analysis between RS and 22 immune cells and found that most immune cells were associated with RS. Infiltration of resting memory CD4+ T cells, monocytes, resting mast cells, B cells, macrophages M1 and M2, etc. was negatively correlated with RS (Figure S2A–G); whereas infiltration of regulatory T cells, activated memory CD4+ T cells, plasma cells, macrophages M0, etc. was positively related to RS (Figure S2H–N). For the rest of immune cells, there was no significant correlations (Figure S2O–V). Subsequently, we quantified the relative abundance of immune cell infiltration with ssGSEA algorithms. As seen in Figure 7A, high‐risk group was enriched with more adaptive immune cells, including activated B cells, CD4^+^ T cells, CD8^+^ T cells, etc.

Immune checkpoint inhibitors (ICIs) have become critical therapies for tumor‐targeted molecular treatments,^31^, ^32^, ^33^ and recent studies have shed light on IPS' role in foreseeing melanoma patients' response to ICI, which was largely dependent on the preexisting high immunogenicity potential.^25^ We have therefore exhaustively investigated the relationship between IPS and the immune profile of KIRC patients. The four scores were intended to estimate a patient's likelihood of accepting ICI treatment. In our experiment, we noted a marked rise in scores in high‐risk group (*p* = 0.036) (Figure 7B; Figure S3A–C). This implied that the high‐risk group seemed to have a stronger immunogenic phenotype. We then observed that high‐risk patients have elevated PD‐1 (*p* = 0.0078) and CTLA‐4 gene expression level (*p* < 0.0001, Figure 7C,D), while low‐risk patients have increased PD‐L1 expression (Figure 7E). These findings pointed to high‐risk patients as promising candidates for ICI treatment.

## 4 DISCUSSION

As the commonest pathological category of renal cell carcinoma (RCC), patients with KIRC have a poorer outcome than other RCC types.^34^ In general, KIRC predictions are largely dependent on cancer pathological stage and TNM stage; yet, these parameters still fall short of precision for early‐stage patients.^35^ Thus, determining biomarkers for early detection and prediction of KIRC survival is essential. With the discovery of tumor markers, the early detection of some malignancies, such as prostate cancer and primary liver cancer, as well as their treatment have been remarkably improved.^36^, ^37^ However, clinically available markers are lacking in KIRC. Advances in genome sequencing and bioinformatics have greatly facilitated the detection of gene sequencing biomarkers that can improve the categorization of cancer and personalized treatment.^38^, ^39^, ^40^

Podocytes are epithelial cells present outside the glomerular capillary fundamentals, and their function and structural integrity directly affect the filtration function of glomerulus.^9^ More than 50 genes, including CD2AP, WT1, RHOA, GLEEP1, and ANLN, have been found to act as podocyte surface markers. Alterations in these markers can affect the architecture and composition of the podocyte, thereby impairing glomerular filtration membrane barrier function.^14^ At present, the majority of research on podocytes are concentrated on podocyte diseases, proteinuria and other disorders,^41^ but in KIRC, relevant studies are rare. We are the first research to explore and verify the potential value of PAGs in KIRC. As such, our study will be inspirational and valuable to other researchers.

In this work, we first analyzed 53 PAGs for CNV and expression data and demonstrated the association of these PAGs with the development and metastasis of KIRC by GO and KEGG enrichment analysis. Then, we randomly divided 518 samples from TCGA‐KIRC into training and internal validation cohort. In training cohort, five genes, WT1, OSGEP, CUBN, ANLN, and RHOA, were selected using Lasso regression and Cox regression analysis, and the formulae for RS were obtained on this basis. Based on five gene expression levels and correlation coefficients, we calculated RS for each KIRC patient and categorized the patients into high‐ and low‐ risk groups dependent on the median RS. In all four cohorts, we found a significant difference in prognosis between the high‐ and low‐risk groups of KIRC patients (*p* ≤ 0.002), with low‐risk group displaying a more positive prognosis. This meant that RS was highly correlated with KIRC prognosis and that RS was probably a negative prognostic agent. Time‐dependent ROC curves were plotted to describe RS' accuracy in forecasting patient outcomes. Typically, AUC > 0.70 meant high predictive diagnostic value. In four cohorts, the AUC of the ROC curve for RS all reached 0.70, which strongly proved the effectiveness of its prediction. Correlation analysis in combination with clinicopathological parameters showed higher RS was more relevant to higher stage, grade and metastasis, and Cox regression demonstrates that RS represented an independent KIRC prognostic factor. Prognostic performance of RS in the KIRC stratified analysis again validated this risk model's hidden values and broadens its scope of use. Moreover, GSEA results showed a significantly reduced enrichment of tumor signaling pathways in low‐risk KIRC patients

Among these five genes, WT1 is mutated and inactivated in Wilm's tumor and causes tumorigenesis, which has a tumor suppressor effect.^42^ Sampson et al. found that WT1 is poorly expressed in adult kidney, but its expression is raised in KIRC. The high expression of WT1 can upregulate E‐cadherin expression and induce tumor cell epithelial‐mesenchymal hybrid transition (EMHT). This is a differentiated state of tumor cells. At this time, cancer cells maintain EMT (epithelial‐mesenchymal transition) and MET (mesenchymal‐epithelial transition) properties, which act in facilitating tumor cell plasticity and tumor progression.^43^ RhoA is a part of the small GTPase rho family and serves as a functional switch in the signaling cascade.^44^ It was found that increasing tumor cell proliferation and invasive capacity by inhibiting RhoA‐ROCK axis.^45^, ^46^ CUBN, acting as an endocytic receptor, is highly specific for expression in KIRC. The increased expression of CUBN suggested a good prognosis for KIRC patients.^47^ ANLN, as actin‐binding protein, is mainly involved with cytoplasmic division.^48^ Dysregulation of ANLN expression has already occurred in various human cancers such as breast and colorectal cancers.^49^ In KIRC, the higher the ANLN expression, the poorer the prognosis of patient.^50^ OSGEP, a member of the KEOPS complex, has been poorly investigated for its role in the progression of KIRC.^15^ The biofunction of these five genes was a partial clue to improving knowledge of the prognostic value of RS, but the potential mechanisms of these genes and RS in KIRC development need to be investigated in depth.

Immune cell infiltration in TIME not only matters for tumor proliferation and metastasis,^51^, ^52^ but also affects the patient's response to immunotherapy.^53^ Chen et al. classified patients into three basic immune profiles based on their response to anti‐PD‐L1/PD‐1 therapy, including immune‐inflamed phenotype, immune‐excluded phenotype, and immune‐desert phenotype.^53^ In conjunction with immune infiltration in TIME, we observed that high‐risk group mainly corresponds to immune‐inflamed phenotype. There are many CD4^+^ T cells and CD8^+^ T cells in this phenotype, accompanied by monocytes and myeloid cells. In clinical treatment, immune‐inflamed phenotype is most responsive to anti‐PD‐L1/PD‐1 therapy. Meanwhile, we found higher expression of PD‐1 and CTLA‐4 genes in high‐risk patients, suggesting that tumor immunogenicity may be stronger in the high‐risk group. Furthermore, it has been demonstrated that tumor mutational burden (TMB) predicts patient response to ICI and clinical benefit, with higher TMB being associated with better immunotherapy outcomes for patients.^54^, ^55^ In our research, TMB was dramatically greater in high‐risk group compared to low‐risk group, although there was no statistical difference (Figure s3D). It also provided side evidence to indicate that the patients in high‐risk group may be better responsive to immunotherapy.

Despite high accuracy of the podocyte‐related prognostic model in predicting KIRC patient prognosis, our study remains limited. First, we mainly used bioinformatics methods to construct the model and lacked some clinical experiments, including PCR, protein blotting, etc. to externally validate the model. Second, we concentrated our study on transcriptome analysis and neglect to consider the impact that epigenetic modifications and other events have on the results. Therefore, the ability of the podocyte‐related prognostic model to predict patient responsiveness to ICIs remains open to debate and requires further clinical trials to validate.

## 5 CONCLUSION

In this study, we developed an in‐depth understanding of PAG's contribution to KIRC and constructed a prognostic model of KIRC from PAG's transcriptomic analysis first. The risk model works effectively in forecasting prognosis and treatment response in KIRC and potentially to improve therapeutic management. Overall, this five‐gene prognostic model may serve as a highly accurate and reliable predictive tool for KIRC.

## CONFLICT OF INTEREST

The authors declare that they have no competing interest.

## AUTHOR CONTRIBUTIONS

Hua‐Guo Xu and Jie‐Xin Zhang conceived the study and were involved in study design, execution, and manuscript revision. Can Chen and Rui‐Xia Yang performed the bioinformatics analysis and manuscript writing. All authors contributed to the article and approved the submitted version.

## ETHICS APPROVAL AND CONSENT TO PARTICIPATE

Not applicable.

## CONSENT FOR PUBLICATION

All authors agree to publish.

## DISCLAIMER

The funder of the study had no role in the study design, data collection, data analysis, data interpretation, or writing of the manuscript. The corresponding author had full access to all the data in the study and has final responsibility for the decision to submit for publication.

## Supporting information


**Appendix S1** Supporting Information

## Data Availability

The data that support the findings of this study are available from the corresponding author upon reasonable request.
