# Supplementary material for: Construction and validation of a prognostic model for kidney renal clear cell carcinoma based on podocyte‐associated genes
Source: Cancer Med. 2022 Apr 4;11(19):3549–62. doi: 10.1002/cam4.4733 (PMC9554457; doi:10.1002/cam4.4733)
Supplement: Supplementary file 1 — Appendix S1 Supporting Information [file CAM4-11-3549-s001.docx]

Supplementary Appendix

This appendix has been provided by the authors to give readers additional information about their work.

Supplement to: Construction and validation of a prognostic model for kidney renal clear cell carcinoma based on podocyte-associated genes

**Construction and validation of a prognostic model for kidney renal clear cell carcinoma based on podocyte-associated genes**

***Supplemental Materials***

**Table of Contents**

**Table S1.** Expression profile of 53 podocyte-associated genes in KIRC and normal tissues

**Table S2.** Results of GO and KEGG pathway analysis

**Table S3.** Clinical information of four cohorts

**Figure S1.** Comparison of our model with other four KIRC models

**Figure S2.** Relationships of risk score with 22 immune cells

**Figure S3.** The IPS and TMB among low-risk and high-risk groups.

**Table S1. Expression profile of 53 podocyte-associated genes in KIRC and normal tissues**

| gene | normal | tumor | logFC | pValue | FDR |
| --- | --- | --- | --- | --- | --- |
| NUP107 | 1.971633 | 2.294601 | 0.218852 | 1.31E-19 | 6.55E-19 |
| NUP85 | 2.009757 | 2.619752 | 0.38241 | 4.95E-30 | 4.95E-29 |
| NUP133 | 3.293249 | 2.98482 | -0.14187 | 4.50E-10 | 1.07E-09 |
| NUP93 | 2.030566 | 1.838276 | -0.14353 | 0.012271 | 0.015339 |
| XPO5 | 2.574943 | 2.470683 | -0.05963 | 0.001809 | 0.002381 |
| WT1 | 2.201232 | 0.688143 | -1.67753 | 1.48E-19 | 6.74E-19 |
| SMARCAL1 | 2.3919 | 2.652774 | 0.149344 | 2.25E-15 | 8.05E-15 |
| SGPL1 | 3.989322 | 3.596778 | -0.14944 | 3.86E-11 | 1.02E-10 |
| SCARB2 | 5.759393 | 5.771008 | 0.002907 | 0.231234 | 0.262766 |
| COQ8B | 2.533824 | 2.876406 | 0.182951 | 1.16E-19 | 6.46E-19 |
| COQ6 | 1.719702 | 1.435405 | -0.2607 | 8.25E-14 | 2.43E-13 |
| COQ2 | 2.045842 | 1.723036 | -0.24774 | 1.59E-14 | 5.29E-14 |
| PDSS2 | 3.523586 | 2.721919 | -0.37242 | 4.56E-33 | 7.60E-32 |
| OSGEP | 2.122866 | 2.480147 | 0.224412 | 1.05E-13 | 2.91E-13 |
| TP53RK | 2.499937 | 2.434972 | -0.03799 | 0.076279 | 0.090809 |
| TPRKB | 2.909174 | 3.290712 | 0.17779 | 2.60E-18 | 1.09E-17 |
| LAGE3 | 3.071442 | 3.145462 | 0.034356 | 0.502633 | 0.534716 |
| WDR73 | 0.629304 | 0.734623 | 0.223247 | 0.000125 | 0.000184 |
| PTPRO | 2.793325 | 1.082366 | -1.36779 | 2.74E-08 | 5.48E-08 |
| EMP2 | 2.573625 | 2.711441 | 0.075258 | 0.013488 | 0.016449 |
| CUBN | 4.131689 | 4.374754 | 0.08247 | 0.773261 | 0.773261 |
| ITGA3 | 4.554817 | 4.960693 | 0.123149 | 1.42E-06 | 2.45E-06 |
| ITGB4 | 2.723488 | 3.04418 | 0.160598 | 0.002579 | 0.003307 |
| LAMB2 | 5.602014 | 5.764471 | 0.041243 | 0.00064 | 0.000865 |
| ACTN4 | 6.786617 | 6.477038 | -0.06736 | 5.25E-09 | 1.14E-08 |
| INF2 | 2.889769 | 4.003227 | 0.470209 | 9.21E-29 | 7.68E-28 |
| MYH9 | 7.089939 | 7.140349 | 0.010221 | 0.210199 | 0.244417 |
| MYO1E | 3.503244 | 3.245605 | -0.1102 | 3.00E-06 | 5.00E-06 |
| AVIL | 0.57735 | 0.837193 | 0.536115 | 4.00E-06 | 6.45E-06 |
| PLCE1 | 1.792126 | 1.341431 | -0.4179 | 6.58E-05 | 9.97E-05 |
| ANLN | 0.458307 | 1.367022 | 1.576651 | 1.83E-30 | 2.28E-29 |
| CRB2 | 1.676438 | 0.422103 | -1.98973 | 9.08E-10 | 2.06E-09 |
| CD2AP | 4.420126 | 3.714491 | -0.25092 | 4.30E-25 | 2.69E-24 |
| NPHS2 | 4.480005 | 0.249848 | -4.16438 | 7.34E-27 | 5.24E-26 |
| NPHS1 | 2.852945 | 0.216475 | -3.72018 | 1.04E-15 | 4.00E-15 |
| FAT1 | 4.456648 | 4.148136 | -0.1035 | 0.000526 | 0.000731 |
| TRPC6 | 0.890548 | 1.234959 | 0.471698 | 3.32E-07 | 6.39E-07 |
| DGKE | 0.97012 | 0.965718 | -0.00656 | 0.49265 | 0.534716 |
| ARHGAP24 | 4.658274 | 3.063187 | -0.60476 | 1.69E-39 | 8.46E-38 |
| ARHGDIA | 5.765799 | 6.064921 | 0.072968 | 6.78E-14 | 2.12E-13 |
| KANK1 | 3.294435 | 2.917364 | -0.17537 | 2.79E-10 | 6.97E-10 |
| ITSN1 | 1.577146 | 1.62583 | 0.043859 | 0.340541 | 0.378379 |
| ITSN2 | 2.736452 | 2.689642 | -0.02489 | 0.704673 | 0.719054 |
| KANK2 | 4.014625 | 4.31794 | 0.105078 | 1.01E-06 | 1.81E-06 |
| MAGI2 | 1.924021 | 0.924149 | -1.05793 | 1.84E-37 | 4.60E-36 |
| TNS2 | 4.122945 | 4.410589 | 0.097296 | 6.44E-06 | 1.01E-05 |
| DLC1 | 2.98021 | 2.887875 | -0.04541 | 0.607538 | 0.632852 |
| RHOA | 7.533 | 7.286546 | -0.04799 | 5.52E-07 | 1.02E-06 |
| RAC1 | 6.407343 | 6.614069 | 0.045812 | 1.29E-08 | 2.69E-08 |
| CDC42 | 6.154516 | 5.967415 | -0.04454 | 0.000162 | 0.000231 |

**Table 2 Results of GO and KEGG pathway analysis**

| **ONTOLOGY** | **ID** | **Description** | **GeneRatio** | **BgRatio** | **pvalue** | **p.adjust** | **qvalue** | **Count** |
| --- | --- | --- | --- | --- | --- | --- | --- | --- |
| BP | GO:0001822 | kidney development | 14/48 | 283/18866 | 6.47E-15 | 6.79E-12 | 4.58E-12 | 14 |
| BP | GO:0072001 | renal system development | 14/48 | 292/18866 | 9.98E-15 | 6.79E-12 | 4.58E-12 | 14 |
| BP | GO:0001655 | urogenital system development | 14/48 | 330/18866 | 5.38E-14 | 1.94E-11 | 1.31E-11 | 14 |
| BP | GO:0072006 | nephron development | 11/48 | 143/18866 | 5.71E-14 | 1.94E-11 | 1.31E-11 | 11 |
| BP | GO:0061318 | renal filtration cell differentiation | 6/48 | 19/18866 | 5.19E-12 | 1.18E-09 | 7.94E-10 | 6 |
| BP | GO:0072112 | glomerular visceral epithelial cell differentiation | 6/48 | 19/18866 | 5.19E-12 | 1.18E-09 | 7.94E-10 | 6 |
| BP | GO:0072311 | glomerular epithelial cell differentiation | 6/48 | 20/18866 | 7.4E-12 | 1.44E-09 | 9.7E-10 | 6 |
| BP | GO:0072010 | glomerular epithelium development | 6/48 | 23/18866 | 1.92E-11 | 3.26E-09 | 2.2E-09 | 6 |
| BP | GO:0007015 | actin filament organization | 13/48 | 434/18866 | 3.92E-11 | 5.93E-09 | 4E-09 | 13 |
| BP | GO:0032835 | glomerulus development | 7/48 | 64/18866 | 2.43E-10 | 3.31E-08 | 2.23E-08 | 7 |
| BP | GO:0051056 | regulation of small GTPase mediated signal transduction | 11/48 | 323/18866 | 4.02E-10 | 4.97E-08 | 3.35E-08 | 11 |
| BP | GO:0031589 | cell-substrate adhesion | 11/48 | 359/18866 | 1.22E-09 | 1.39E-07 | 9.36E-08 | 11 |
| BP | GO:0035850 | epithelial cell differentiation involved in kidney development | 6/48 | 47/18866 | 1.95E-09 | 2E-07 | 1.35E-07 | 6 |
| BP | GO:0097581 | lamellipodium organization | 7/48 | 87/18866 | 2.19E-09 | 2E-07 | 1.35E-07 | 7 |
| BP | GO:0007266 | Rho protein signal transduction | 8/48 | 140/18866 | 2.21E-09 | 2E-07 | 1.35E-07 | 8 |
| BP | GO:0031346 | positive regulation of cell projection organization | 11/48 | 394/18866 | 3.24E-09 | 2.72E-07 | 1.83E-07 | 11 |
| BP | GO:0010810 | regulation of cell-substrate adhesion | 9/48 | 217/18866 | 3.39E-09 | 2.72E-07 | 1.83E-07 | 9 |
| BP | GO:0032970 | regulation of actin filament-based process | 11/48 | 405/18866 | 4.32E-09 | 3.26E-07 | 2.2E-07 | 11 |
| BP | GO:0061005 | cell differentiation involved in kidney development | 6/48 | 57/18866 | 6.46E-09 | 4.62E-07 | 3.12E-07 | 6 |
| BP | GO:0032836 | glomerular basement membrane development | 4/48 | 10/18866 | 7.66E-09 | 5.21E-07 | 3.51E-07 | 4 |
| BP | GO:0007265 | Ras protein signal transduction | 10/48 | 346/18866 | 1.33E-08 | 8.63E-07 | 5.82E-07 | 10 |
| BP | GO:0072015 | glomerular visceral epithelial cell development | 4/48 | 12/18866 | 1.8E-08 | 1.11E-06 | 7.5E-07 | 4 |
| BP | GO:0032956 | regulation of actin cytoskeleton organization | 10/48 | 360/18866 | 1.94E-08 | 1.15E-06 | 7.74E-07 | 10 |
| BP | GO:0030032 | lamellipodium assembly | 6/48 | 69/18866 | 2.08E-08 | 1.18E-06 | 7.97E-07 | 6 |
| BP | GO:0072310 | glomerular epithelial cell development | 4/48 | 13/18866 | 2.59E-08 | 1.41E-06 | 9.52E-07 | 4 |
| BP | GO:0046578 | regulation of Ras protein signal transduction | 8/48 | 194/18866 | 2.87E-08 | 1.5E-06 | 1.01E-06 | 8 |
| BP | GO:0022604 | regulation of cell morphogenesis | 11/48 | 499/18866 | 3.7E-08 | 1.87E-06 | 1.26E-06 | 11 |
| BP | GO:0072073 | kidney epithelium development | 7/48 | 142/18866 | 6.71E-08 | 3.26E-06 | 2.2E-06 | 7 |
| BP | GO:0035023 | regulation of Rho protein signal transduction | 6/48 | 86/18866 | 7.91E-08 | 3.71E-06 | 2.5E-06 | 6 |
| BP | GO:0070525 | tRNA threonylcarbamoyladenosine metabolic process | 4/48 | 17/18866 | 8.57E-08 | 3.89E-06 | 2.62E-06 | 4 |
| BP | GO:0006744 | ubiquinone biosynthetic process | 4/48 | 18/18866 | 1.1E-07 | 4.68E-06 | 3.15E-06 | 4 |
| BP | GO:1901663 | quinone biosynthetic process | 4/48 | 18/18866 | 1.1E-07 | 4.68E-06 | 3.15E-06 | 4 |
| BP | GO:1902743 | regulation of lamellipodium organization | 5/48 | 50/18866 | 1.67E-07 | 6.74E-06 | 4.55E-06 | 5 |
| BP | GO:0010771 | negative regulation of cell morphogenesis involved in differentiation | 6/48 | 98/18866 | 1.73E-07 | 6.74E-06 | 4.55E-06 | 6 |
| BP | GO:0006743 | ubiquinone metabolic process | 4/48 | 20/18866 | 1.73E-07 | 6.74E-06 | 4.55E-06 | 4 |
| BP | GO:0034446 | substrate adhesion-dependent cell spreading | 6/48 | 106/18866 | 2.76E-07 | 1.04E-05 | 7.05E-06 | 6 |
| BP | GO:0072009 | nephron epithelium development | 6/48 | 110/18866 | 3.44E-07 | 1.27E-05 | 8.54E-06 | 6 |
| BP | GO:0120032 | regulation of plasma membrane bounded cell projection assembly | 7/48 | 183/18866 | 3.79E-07 | 1.36E-05 | 9.15E-06 | 7 |
| BP | GO:0051058 | negative regulation of small GTPase mediated signal transduction | 5/48 | 59/18866 | 3.89E-07 | 1.36E-05 | 9.15E-06 | 5 |
| BP | GO:0060491 | regulation of cell projection assembly | 7/48 | 185/18866 | 4.08E-07 | 1.39E-05 | 9.36E-06 | 7 |
| BP | GO:0010592 | positive regulation of lamellipodium assembly | 4/48 | 25/18866 | 4.49E-07 | 1.41E-05 | 9.5E-06 | 4 |
| BP | GO:0097205 | renal filtration | 4/48 | 25/18866 | 4.49E-07 | 1.41E-05 | 9.5E-06 | 4 |
| BP | GO:0031345 | negative regulation of cell projection organization | 7/48 | 188/18866 | 4.55E-07 | 1.41E-05 | 9.5E-06 | 7 |
| BP | GO:0110053 | regulation of actin filament organization | 8/48 | 278/18866 | 4.55E-07 | 1.41E-05 | 9.5E-06 | 8 |
| BP | GO:0010812 | negative regulation of cell-substrate adhesion | 5/48 | 68/18866 | 7.95E-07 | 2.41E-05 | 1.62E-05 | 5 |
| BP | GO:0010769 | regulation of cell morphogenesis involved in differentiation | 8/48 | 310/18866 | 1.03E-06 | 3.06E-05 | 2.07E-05 | 8 |
| BP | GO:1902745 | positive regulation of lamellipodium organization | 4/48 | 33/18866 | 1.43E-06 | 4.14E-05 | 2.79E-05 | 4 |
| BP | GO:0006409 | tRNA export from nucleus | 4/48 | 34/18866 | 1.62E-06 | 4.49E-05 | 3.03E-05 | 4 |
| BP | GO:0071431 | tRNA-containing ribonucleoprotein complex export from nucleus | 4/48 | 34/18866 | 1.62E-06 | 4.49E-05 | 3.03E-05 | 4 |
| BP | GO:1901661 | quinone metabolic process | 4/48 | 36/18866 | 2.05E-06 | 5.57E-05 | 3.76E-05 | 4 |
| BP | GO:0051031 | tRNA transport | 4/48 | 37/18866 | 2.29E-06 | 6.11E-05 | 4.12E-05 | 4 |
| BP | GO:0010591 | regulation of lamellipodium assembly | 4/48 | 38/18866 | 2.55E-06 | 6.56E-05 | 4.42E-05 | 4 |
| BP | GO:0097064 | ncRNA export from nucleus | 4/48 | 38/18866 | 2.55E-06 | 6.56E-05 | 4.42E-05 | 4 |
| BP | GO:0043470 | regulation of carbohydrate catabolic process | 5/48 | 90/18866 | 3.22E-06 | 8.11E-05 | 5.47E-05 | 5 |
| BP | GO:1902903 | regulation of supramolecular fiber organization | 8/48 | 373/18866 | 4.1E-06 | 0.000101 | 6.84E-05 | 8 |
| BP | GO:0042181 | ketone biosynthetic process | 4/48 | 44/18866 | 4.65E-06 | 0.000112 | 7.56E-05 | 4 |
| BP | GO:0045666 | positive regulation of neuron differentiation | 8/48 | 380/18866 | 4.7E-06 | 0.000112 | 7.56E-05 | 8 |
| BP | GO:0006999 | nuclear pore organization | 3/48 | 14/18866 | 5.52E-06 | 0.000129 | 8.73E-05 | 3 |
| BP | GO:0120034 | positive regulation of plasma membrane bounded cell projection assembly | 5/48 | 104/18866 | 6.55E-06 | 0.000151 | 0.000102 | 5 |
| BP | GO:0031532 | actin cytoskeleton reorganization | 5/48 | 105/18866 | 6.87E-06 | 0.000156 | 0.000105 | 5 |
| BP | GO:0007229 | integrin-mediated signaling pathway | 5/48 | 108/18866 | 7.88E-06 | 0.000176 | 0.000119 | 5 |
| BP | GO:0010976 | positive regulation of neuron projection development | 7/48 | 290/18866 | 8.1E-06 | 0.000178 | 0.00012 | 7 |
| BP | GO:0030041 | actin filament polymerization | 6/48 | 193/18866 | 9.1E-06 | 0.000194 | 0.000131 | 6 |
| BP | GO:0046580 | negative regulation of Ras protein signal transduction | 4/48 | 52/18866 | 9.13E-06 | 0.000194 | 0.000131 | 4 |
| BP | GO:1900024 | regulation of substrate adhesion-dependent cell spreading | 4/48 | 53/18866 | 9.85E-06 | 0.000206 | 0.000139 | 4 |
| BP | GO:0031032 | actomyosin structure organization | 6/48 | 200/18866 | 1.12E-05 | 0.00023 | 0.000155 | 6 |
| BP | GO:0034329 | cell junction assembly | 8/48 | 434/18866 | 1.24E-05 | 0.000252 | 0.00017 | 8 |
| BP | GO:0060147 | regulation of posttranscriptional gene silencing | 5/48 | 120/18866 | 1.32E-05 | 0.000257 | 0.000173 | 5 |
| BP | GO:0060966 | regulation of gene silencing by RNA | 5/48 | 120/18866 | 1.32E-05 | 0.000257 | 0.000173 | 5 |
| BP | GO:0075733 | intracellular transport of virus | 4/48 | 57/18866 | 1.32E-05 | 0.000257 | 0.000173 | 4 |
| BP | GO:0003014 | renal system process | 5/48 | 125/18866 | 1.61E-05 | 0.000304 | 0.000205 | 5 |
| BP | GO:1903578 | regulation of ATP metabolic process | 5/48 | 125/18866 | 1.61E-05 | 0.000304 | 0.000205 | 5 |
| BP | GO:0007163 | establishment or maintenance of cell polarity | 6/48 | 220/18866 | 1.92E-05 | 0.000357 | 0.000241 | 6 |
| BP | GO:0008154 | actin polymerization or depolymerization | 6/48 | 221/18866 | 1.96E-05 | 0.000361 | 0.000244 | 6 |
| BP | GO:0046794 | transport of virus | 4/48 | 64/18866 | 2.09E-05 | 0.00038 | 0.000256 | 4 |
| BP | GO:0035024 | negative regulation of Rho protein signal transduction | 3/48 | 22/18866 | 2.3E-05 | 0.000412 | 0.000278 | 3 |
| BP | GO:0044766 | multi-organism transport | 4/48 | 66/18866 | 2.37E-05 | 0.000413 | 0.000279 | 4 |
| BP | GO:1902579 | multi-organism localization | 4/48 | 66/18866 | 2.37E-05 | 0.000413 | 0.000279 | 4 |
| BP | GO:0007160 | cell-matrix adhesion | 6/48 | 230/18866 | 2.46E-05 | 0.000424 | 0.000286 | 6 |
| BP | GO:0032271 | regulation of protein polymerization | 6/48 | 231/18866 | 2.52E-05 | 0.000429 | 0.000289 | 6 |
| BP | GO:0001667 | ameboidal-type cell migration | 8/48 | 481/18866 | 2.59E-05 | 0.000436 | 0.000294 | 8 |
| BP | GO:0050769 | positive regulation of neurogenesis | 8/48 | 485/18866 | 2.75E-05 | 0.000455 | 0.000307 | 8 |
| BP | GO:0060968 | regulation of gene silencing | 5/48 | 140/18866 | 2.78E-05 | 0.000455 | 0.000307 | 5 |
| BP | GO:0003094 | glomerular filtration | 3/48 | 24/18866 | 3.01E-05 | 0.000488 | 0.000329 | 3 |
| BP | GO:0006405 | RNA export from nucleus | 5/48 | 144/18866 | 3.18E-05 | 0.000509 | 0.000343 | 5 |
| BP | GO:0003071 | renal system process involved in regulation of systemic arterial blood pressure | 3/48 | 25/18866 | 3.42E-05 | 0.000535 | 0.000361 | 3 |
| BP | GO:0046339 | diacylglycerol metabolic process | 3/48 | 25/18866 | 3.42E-05 | 0.000535 | 0.000361 | 3 |
| BP | GO:0010631 | epithelial cell migration | 7/48 | 365/18866 | 3.56E-05 | 0.000551 | 0.000372 | 7 |
| BP | GO:0090132 | epithelium migration | 7/48 | 368/18866 | 3.75E-05 | 0.000574 | 0.000387 | 7 |
| BP | GO:0090130 | tissue migration | 7/48 | 374/18866 | 4.16E-05 | 0.000629 | 0.000424 | 7 |
| BP | GO:0010977 | negative regulation of neuron projection development | 5/48 | 154/18866 | 4.38E-05 | 0.000655 | 0.000442 | 5 |
| BP | GO:0051017 | actin filament bundle assembly | 5/48 | 157/18866 | 4.8E-05 | 0.000705 | 0.000475 | 5 |
| BP | GO:1900034 | regulation of cellular response to heat | 4/48 | 79/18866 | 4.82E-05 | 0.000705 | 0.000475 | 4 |
| BP | GO:0006110 | regulation of glycolytic process | 4/48 | 80/18866 | 5.06E-05 | 0.000733 | 0.000494 | 4 |
| BP | GO:0061572 | actin filament bundle organization | 5/48 | 161/18866 | 5.41E-05 | 0.000776 | 0.000523 | 5 |
| BP | GO:0016925 | protein sumoylation | 4/48 | 82/18866 | 5.58E-05 | 0.000782 | 0.000528 | 4 |
| BP | GO:0043467 | regulation of generation of precursor metabolites and energy | 5/48 | 162/18866 | 5.58E-05 | 0.000782 | 0.000528 | 5 |
| BP | GO:0001570 | vasculogenesis | 4/48 | 83/18866 | 5.85E-05 | 0.000812 | 0.000548 | 4 |
| BP | GO:0030833 | regulation of actin filament polymerization | 5/48 | 174/18866 | 7.83E-05 | 0.001076 | 0.000726 | 5 |
| BP | GO:0051492 | regulation of stress fiber assembly | 4/48 | 91/18866 | 8.38E-05 | 0.00114 | 0.000769 | 4 |
| BP | GO:0006611 | protein export from nucleus | 5/48 | 184/18866 | 0.000102 | 0.001373 | 0.000926 | 5 |
| BP | GO:0051258 | protein polymerization | 6/48 | 300/18866 | 0.000108 | 0.001436 | 0.000969 | 6 |
| BP | GO:0007162 | negative regulation of cell adhesion | 6/48 | 301/18866 | 0.00011 | 0.001448 | 0.000977 | 6 |
| BP | GO:0007044 | cell-substrate junction assembly | 4/48 | 99/18866 | 0.000116 | 0.001522 | 0.001026 | 4 |
| BP | GO:0008064 | regulation of actin polymerization or depolymerization | 5/48 | 190/18866 | 0.000118 | 0.001535 | 0.001035 | 5 |
| BP | GO:0030832 | regulation of actin filament length | 5/48 | 191/18866 | 0.000121 | 0.001559 | 0.001051 | 5 |
| BP | GO:0110020 | regulation of actomyosin structure organization | 4/48 | 102/18866 | 0.000131 | 0.001624 | 0.001095 | 4 |
| BP | GO:0150115 | cell-substrate junction organization | 4/48 | 102/18866 | 0.000131 | 0.001624 | 0.001095 | 4 |
| BP | GO:0043254 | regulation of protein-containing complex assembly | 7/48 | 449/18866 | 0.000131 | 0.001624 | 0.001095 | 7 |
| BP | GO:0032506 | cytokinetic process | 3/48 | 39/18866 | 0.000132 | 0.001624 | 0.001095 | 3 |
| BP | GO:2000249 | regulation of actin cytoskeleton reorganization | 3/48 | 39/18866 | 0.000132 | 0.001624 | 0.001095 | 3 |
| BP | GO:0032231 | regulation of actin filament bundle assembly | 4/48 | 104/18866 | 0.000141 | 0.00171 | 0.001153 | 4 |
| BP | GO:0016052 | carbohydrate catabolic process | 5/48 | 200/18866 | 0.000151 | 0.001762 | 0.001188 | 5 |
| BP | GO:0050657 | nucleic acid transport | 5/48 | 200/18866 | 0.000151 | 0.001762 | 0.001188 | 5 |
| BP | GO:0050658 | RNA transport | 5/48 | 200/18866 | 0.000151 | 0.001762 | 0.001188 | 5 |
| BP | GO:0030038 | contractile actin filament bundle assembly | 4/48 | 106/18866 | 0.000151 | 0.001762 | 0.001188 | 4 |
| BP | GO:0043149 | stress fiber assembly | 4/48 | 106/18866 | 0.000151 | 0.001762 | 0.001188 | 4 |
| BP | GO:0051236 | establishment of RNA localization | 5/48 | 203/18866 | 0.000161 | 0.001862 | 0.001256 | 5 |
| BP | GO:0060071 | Wnt signaling pathway, planar cell polarity pathway | 4/48 | 108/18866 | 0.000163 | 0.001862 | 0.001256 | 4 |
| BP | GO:0051168 | nuclear export | 5/48 | 204/18866 | 0.000165 | 0.001873 | 0.001263 | 5 |
| BP | GO:0090175 | regulation of establishment of planar polarity | 4/48 | 110/18866 | 0.000175 | 0.001952 | 0.001317 | 4 |
| BP | GO:0006898 | receptor-mediated endocytosis | 6/48 | 328/18866 | 0.000175 | 0.001952 | 0.001317 | 6 |
| BP | GO:0006109 | regulation of carbohydrate metabolic process | 5/48 | 210/18866 | 0.000189 | 0.002055 | 0.001386 | 5 |
| BP | GO:0030100 | regulation of endocytosis | 5/48 | 210/18866 | 0.000189 | 0.002055 | 0.001386 | 5 |
| BP | GO:0045197 | establishment or maintenance of epithelial cell apical/basal polarity | 3/48 | 44/18866 | 0.00019 | 0.002055 | 0.001386 | 3 |
| BP | GO:0097178 | ruffle assembly | 3/48 | 44/18866 | 0.00019 | 0.002055 | 0.001386 | 3 |
| BP | GO:0006406 | mRNA export from nucleus | 4/48 | 113/18866 | 0.000194 | 0.00206 | 0.001389 | 4 |
| BP | GO:0071427 | mRNA-containing ribonucleoprotein complex export from nucleus | 4/48 | 113/18866 | 0.000194 | 0.00206 | 0.001389 | 4 |
| BP | GO:0006096 | glycolytic process | 4/48 | 115/18866 | 0.000207 | 0.002187 | 0.001475 | 4 |
| BP | GO:0006757 | ATP generation from ADP | 4/48 | 116/18866 | 0.000214 | 0.002226 | 0.001501 | 4 |
| BP | GO:0060964 | regulation of gene silencing by miRNA | 4/48 | 116/18866 | 0.000214 | 0.002226 | 0.001501 | 4 |
| BP | GO:0019058 | viral life cycle | 6/48 | 341/18866 | 0.000216 | 0.002226 | 0.001502 | 6 |
| BP | GO:0010721 | negative regulation of cell development | 6/48 | 343/18866 | 0.000223 | 0.00228 | 0.001538 | 6 |
| BP | GO:0002064 | epithelial cell development | 5/48 | 221/18866 | 0.00024 | 0.002433 | 0.001641 | 5 |
| BP | GO:0035088 | establishment or maintenance of apical/basal cell polarity | 3/48 | 48/18866 | 0.000247 | 0.002469 | 0.001665 | 3 |
| BP | GO:0061245 | establishment or maintenance of bipolar cell polarity | 3/48 | 48/18866 | 0.000247 | 0.002469 | 0.001665 | 3 |
| BP | GO:1900542 | regulation of purine nucleotide metabolic process | 4/48 | 121/18866 | 0.000252 | 0.002502 | 0.001688 | 4 |
| BP | GO:0010811 | positive regulation of cell-substrate adhesion | 4/48 | 122/18866 | 0.00026 | 0.002548 | 0.001719 | 4 |
| BP | GO:0045665 | negative regulation of neuron differentiation | 5/48 | 225/18866 | 0.00026 | 0.002548 | 0.001719 | 5 |
| BP | GO:0006140 | regulation of nucleotide metabolic process | 4/48 | 123/18866 | 0.000268 | 0.002607 | 0.001758 | 4 |
| BP | GO:0001736 | establishment of planar polarity | 4/48 | 124/18866 | 0.000277 | 0.002633 | 0.001775 | 4 |
| BP | GO:0007164 | establishment of tissue polarity | 4/48 | 124/18866 | 0.000277 | 0.002633 | 0.001775 | 4 |
| BP | GO:0046031 | ADP metabolic process | 4/48 | 124/18866 | 0.000277 | 0.002633 | 0.001775 | 4 |
| BP | GO:0001952 | regulation of cell-matrix adhesion | 4/48 | 125/18866 | 0.000285 | 0.002677 | 0.001805 | 4 |
| BP | GO:0034605 | cellular response to heat | 4/48 | 125/18866 | 0.000285 | 0.002677 | 0.001805 | 4 |
| BP | GO:0051496 | positive regulation of stress fiber assembly | 3/48 | 52/18866 | 0.000313 | 0.002917 | 0.001968 | 3 |
| BP | GO:0006403 | RNA localization | 5/48 | 236/18866 | 0.000324 | 0.003002 | 0.002025 | 5 |
| BP | GO:0008033 | tRNA processing | 4/48 | 130/18866 | 0.000331 | 0.003025 | 0.00204 | 4 |
| BP | GO:0071426 | ribonucleoprotein complex export from nucleus | 4/48 | 130/18866 | 0.000331 | 0.003025 | 0.00204 | 4 |
| BP | GO:0071166 | ribonucleoprotein complex localization | 4/48 | 131/18866 | 0.000341 | 0.003076 | 0.002075 | 4 |
| BP | GO:0003093 | regulation of glomerular filtration | 2/48 | 11/18866 | 0.000344 | 0.003076 | 0.002075 | 2 |
| BP | GO:0032060 | bleb assembly | 2/48 | 11/18866 | 0.000344 | 0.003076 | 0.002075 | 2 |
| BP | GO:0006165 | nucleoside diphosphate phosphorylation | 4/48 | 133/18866 | 0.000361 | 0.003192 | 0.002153 | 4 |
| BP | GO:0006997 | nucleus organization | 4/48 | 133/18866 | 0.000361 | 0.003192 | 0.002153 | 4 |
| BP | GO:0046939 | nucleotide phosphorylation | 4/48 | 135/18866 | 0.000382 | 0.003335 | 0.002249 | 4 |
| BP | GO:0099024 | plasma membrane invagination | 4/48 | 135/18866 | 0.000382 | 0.003335 | 0.002249 | 4 |
| BP | GO:0031529 | ruffle organization | 3/48 | 56/18866 | 0.00039 | 0.003365 | 0.002269 | 3 |
| BP | GO:0009135 | purine nucleoside diphosphate metabolic process | 4/48 | 136/18866 | 0.000393 | 0.003365 | 0.002269 | 4 |
| BP | GO:0009179 | purine ribonucleoside diphosphate metabolic process | 4/48 | 136/18866 | 0.000393 | 0.003365 | 0.002269 | 4 |
| BP | GO:0032535 | regulation of cellular component size | 6/48 | 383/18866 | 0.000402 | 0.003419 | 0.002306 | 6 |
| BP | GO:0009185 | ribonucleoside diphosphate metabolic process | 4/48 | 139/18866 | 0.000427 | 0.00361 | 0.002434 | 4 |
| BP | GO:0030010 | establishment of cell polarity | 4/48 | 141/18866 | 0.000451 | 0.003786 | 0.002554 | 4 |
| BP | GO:2000027 | regulation of animal organ morphogenesis | 5/48 | 254/18866 | 0.000454 | 0.003791 | 0.002556 | 5 |
| BP | GO:0031333 | negative regulation of protein-containing complex assembly | 4/48 | 142/18866 | 0.000463 | 0.003842 | 0.002591 | 4 |
| BP | GO:0030198 | extracellular matrix organization | 6/48 | 395/18866 | 0.000473 | 0.003865 | 0.002607 | 6 |
| BP | GO:0010324 | membrane invagination | 4/48 | 143/18866 | 0.000475 | 0.003865 | 0.002607 | 4 |
| BP | GO:0030865 | cortical cytoskeleton organization | 3/48 | 60/18866 | 0.000478 | 0.003865 | 0.002607 | 3 |
| BP | GO:0043062 | extracellular structure organization | 6/48 | 396/18866 | 0.00048 | 0.003865 | 0.002607 | 6 |
| BP | GO:0048548 | regulation of pinocytosis | 2/48 | 13/18866 | 0.000486 | 0.003865 | 0.002607 | 2 |
| BP | GO:1900025 | negative regulation of substrate adhesion-dependent cell spreading | 2/48 | 13/18866 | 0.000486 | 0.003865 | 0.002607 | 2 |
| BP | GO:1905244 | regulation of modification of synaptic structure | 2/48 | 13/18866 | 0.000486 | 0.003865 | 0.002607 | 2 |
| BP | GO:0001738 | morphogenesis of a polarized epithelium | 4/48 | 145/18866 | 0.000501 | 0.003964 | 0.002673 | 4 |
| BP | GO:0051271 | negative regulation of cellular component movement | 6/48 | 400/18866 | 0.000506 | 0.003979 | 0.002684 | 6 |
| BP | GO:0015931 | nucleobase-containing compound transport | 5/48 | 261/18866 | 0.000514 | 0.004019 | 0.00271 | 5 |
| BP | GO:0032233 | positive regulation of actin filament bundle assembly | 3/48 | 62/18866 | 0.000526 | 0.004092 | 0.002759 | 3 |
| BP | GO:0051893 | regulation of focal adhesion assembly | 3/48 | 63/18866 | 0.000551 | 0.00424 | 0.00286 | 3 |
| BP | GO:0090109 | regulation of cell-substrate junction assembly | 3/48 | 63/18866 | 0.000551 | 0.00424 | 0.00286 | 3 |
| BP | GO:0035567 | non-canonical Wnt signaling pathway | 4/48 | 151/18866 | 0.000584 | 0.004462 | 0.003009 | 4 |
| BP | GO:0006090 | pyruvate metabolic process | 4/48 | 152/18866 | 0.000598 | 0.004523 | 0.003051 | 4 |
| BP | GO:0051028 | mRNA transport | 4/48 | 152/18866 | 0.000598 | 0.004523 | 0.003051 | 4 |
| BP | GO:0030837 | negative regulation of actin filament polymerization | 3/48 | 65/18866 | 0.000604 | 0.004544 | 0.003065 | 3 |
| BP | GO:0030048 | actin filament-based movement | 4/48 | 153/18866 | 0.000613 | 0.004585 | 0.003092 | 4 |
| BP | GO:0150116 | regulation of cell-substrate junction organization | 3/48 | 66/18866 | 0.000632 | 0.0047 | 0.00317 | 3 |
| BP | GO:0009132 | nucleoside diphosphate metabolic process | 4/48 | 155/18866 | 0.000644 | 0.004762 | 0.003212 | 4 |
| BP | GO:0045198 | establishment of epithelial cell apical/basal polarity | 2/48 | 15/18866 | 0.000652 | 0.004794 | 0.003233 | 2 |
| BP | GO:0008360 | regulation of cell shape | 4/48 | 156/18866 | 0.000659 | 0.0048 | 0.003237 | 4 |
| BP | GO:1902904 | negative regulation of supramolecular fiber organization | 4/48 | 156/18866 | 0.000659 | 0.0048 | 0.003237 | 4 |
| BP | GO:0010770 | positive regulation of cell morphogenesis involved in differentiation | 4/48 | 157/18866 | 0.000675 | 0.00489 | 0.003298 | 4 |
| BP | GO:0001977 | renal system process involved in regulation of blood volume | 2/48 | 16/18866 | 0.000744 | 0.005298 | 0.003573 | 2 |
| BP | GO:0051014 | actin filament severing | 2/48 | 16/18866 | 0.000744 | 0.005298 | 0.003573 | 2 |
| BP | GO:0072224 | metanephric glomerulus development | 2/48 | 16/18866 | 0.000744 | 0.005298 | 0.003573 | 2 |
| BP | GO:0050771 | negative regulation of axonogenesis | 3/48 | 70/18866 | 0.000751 | 0.005321 | 0.003589 | 3 |
| BP | GO:0050808 | synapse organization | 6/48 | 433/18866 | 0.000766 | 0.005404 | 0.003644 | 6 |
| BP | GO:0051494 | negative regulation of cytoskeleton organization | 4/48 | 163/18866 | 0.000777 | 0.005453 | 0.003677 | 4 |
| BP | GO:0009408 | response to heat | 4/48 | 166/18866 | 0.000832 | 0.005807 | 0.003916 | 4 |
| BP | GO:0050768 | negative regulation of neurogenesis | 5/48 | 295/18866 | 0.000893 | 0.006198 | 0.00418 | 5 |
| BP | GO:0099010 | modification of postsynaptic structure | 2/48 | 18/18866 | 0.000945 | 0.006527 | 0.004402 | 2 |
| BP | GO:0000910 | cytokinesis | 4/48 | 172/18866 | 0.00095 | 0.006527 | 0.004402 | 4 |
| BP | GO:0030050 | vesicle transport along actin filament | 2/48 | 19/18866 | 0.001054 | 0.007175 | 0.004839 | 2 |
| BP | GO:0035089 | establishment of apical/basal cell polarity | 2/48 | 19/18866 | 0.001054 | 0.007175 | 0.004839 | 2 |
| BP | GO:0032272 | negative regulation of protein polymerization | 3/48 | 79/18866 | 0.001067 | 0.007226 | 0.004874 | 3 |
| BP | GO:0019083 | viral transcription | 4/48 | 178/18866 | 0.001078 | 0.007265 | 0.0049 | 4 |
| BP | GO:0046034 | ATP metabolic process | 5/48 | 311/18866 | 0.00113 | 0.007573 | 0.005108 | 5 |
| BP | GO:1905330 | regulation of morphogenesis of an epithelium | 4/48 | 181/18866 | 0.001147 | 0.007653 | 0.005162 | 4 |
| BP | GO:0046931 | pore complex assembly | 2/48 | 20/18866 | 0.00117 | 0.007765 | 0.005237 | 2 |
| BP | GO:0048041 | focal adhesion assembly | 3/48 | 83/18866 | 0.001231 | 0.008134 | 0.005486 | 3 |
| BP | GO:0051961 | negative regulation of nervous system development | 5/48 | 319/18866 | 0.001264 | 0.008301 | 0.005598 | 5 |
| BP | GO:0006399 | tRNA metabolic process | 4/48 | 186/18866 | 0.001269 | 0.008301 | 0.005598 | 4 |
| BP | GO:0061162 | establishment of monopolar cell polarity | 2/48 | 21/18866 | 0.001291 | 0.008365 | 0.005641 | 2 |
| BP | GO:0099515 | actin filament-based transport | 2/48 | 21/18866 | 0.001291 | 0.008365 | 0.005641 | 2 |
| BP | GO:0048013 | ephrin receptor signaling pathway | 3/48 | 87/18866 | 0.00141 | 0.009057 | 0.006108 | 3 |
| BP | GO:0030859 | polarized epithelial cell differentiation | 2/48 | 22/18866 | 0.001417 | 0.009057 | 0.006108 | 2 |
| BP | GO:0061339 | establishment or maintenance of monopolar cell polarity | 2/48 | 22/18866 | 0.001417 | 0.009057 | 0.006108 | 2 |
| BP | GO:0016049 | cell growth | 6/48 | 490/18866 | 0.001449 | 0.009216 | 0.006215 | 6 |
| BP | GO:0019080 | viral gene expression | 4/48 | 195/18866 | 0.001509 | 0.009555 | 0.006444 | 4 |
| BP | GO:0006907 | pinocytosis | 2/48 | 23/18866 | 0.00155 | 0.009766 | 0.006586 | 2 |
| BP | GO:0099563 | modification of synaptic structure | 2/48 | 24/18866 | 0.001688 | 0.010587 | 0.00714 | 2 |
| BP | GO:0007596 | blood coagulation | 5/48 | 343/18866 | 0.00174 | 0.010864 | 0.007327 | 5 |
| BP | GO:0003073 | regulation of systemic arterial blood pressure | 3/48 | 95/18866 | 0.001816 | 0.011283 | 0.00761 | 3 |
| BP | GO:0007599 | hemostasis | 5/48 | 348/18866 | 0.001854 | 0.011469 | 0.007735 | 5 |
| BP | GO:0050817 | coagulation | 5/48 | 349/18866 | 0.001877 | 0.011561 | 0.007797 | 5 |
| BP | GO:1902905 | positive regulation of supramolecular fiber organization | 4/48 | 208/18866 | 0.001911 | 0.011717 | 0.007902 | 4 |
| BP | GO:0045216 | cell-cell junction organization | 4/48 | 210/18866 | 0.001979 | 0.012057 | 0.008132 | 4 |
| BP | GO:0048010 | vascular endothelial growth factor receptor signaling pathway | 3/48 | 98/18866 | 0.001984 | 0.012057 | 0.008132 | 3 |
| BP | GO:0006913 | nucleocytoplasmic transport | 5/48 | 354/18866 | 0.001998 | 0.012083 | 0.008149 | 5 |
| BP | GO:0051169 | nuclear transport | 5/48 | 357/18866 | 0.002072 | 0.01248 | 0.008417 | 5 |
| BP | GO:0008585 | female gonad development | 3/48 | 100/18866 | 0.002102 | 0.012549 | 0.008463 | 3 |
| BP | GO:0045807 | positive regulation of endocytosis | 3/48 | 100/18866 | 0.002102 | 0.012549 | 0.008463 | 3 |
| BP | GO:0034698 | response to gonadotropin | 2/48 | 27/18866 | 0.002136 | 0.012642 | 0.008526 | 2 |
| BP | GO:0072207 | metanephric epithelium development | 2/48 | 27/18866 | 0.002136 | 0.012642 | 0.008526 | 2 |
| BP | GO:0008299 | isoprenoid biosynthetic process | 2/48 | 28/18866 | 0.002297 | 0.013533 | 0.009127 | 2 |
| BP | GO:0046545 | development of primary female sexual characteristics | 3/48 | 105/18866 | 0.002416 | 0.01411 | 0.009516 | 3 |
| BP | GO:0048259 | regulation of receptor-mediated endocytosis | 3/48 | 105/18866 | 0.002416 | 0.01411 | 0.009516 | 3 |
| BP | GO:0090162 | establishment of epithelial cell polarity | 2/48 | 30/18866 | 0.002635 | 0.015195 | 0.010248 | 2 |
| BP | GO:0120033 | negative regulation of plasma membrane bounded cell projection assembly | 2/48 | 30/18866 | 0.002635 | 0.015195 | 0.010248 | 2 |
| BP | GO:1900027 | regulation of ruffle assembly | 2/48 | 30/18866 | 0.002635 | 0.015195 | 0.010248 | 2 |
| BP | GO:0051495 | positive regulation of cytoskeleton organization | 4/48 | 230/18866 | 0.002751 | 0.015797 | 0.010654 | 4 |
| BP | GO:0009266 | response to temperature stimulus | 4/48 | 233/18866 | 0.002882 | 0.016482 | 0.011116 | 4 |
| BP | GO:0006275 | regulation of DNA replication | 3/48 | 112/18866 | 0.002901 | 0.016518 | 0.01114 | 3 |
| BP | GO:0044319 | wound healing, spreading of cells | 2/48 | 33/18866 | 0.003183 | 0.017899 | 0.012072 | 2 |
| BP | GO:0046627 | negative regulation of insulin receptor signaling pathway | 2/48 | 33/18866 | 0.003183 | 0.017899 | 0.012072 | 2 |
| BP | GO:0090505 | epiboly involved in wound healing | 2/48 | 33/18866 | 0.003183 | 0.017899 | 0.012072 | 2 |
| BP | GO:0040013 | negative regulation of locomotion | 5/48 | 397/18866 | 0.003277 | 0.018353 | 0.012378 | 5 |
| BP | GO:0090504 | epiboly | 2/48 | 34/18866 | 0.003376 | 0.018831 | 0.0127 | 2 |
| BP | GO:0009150 | purine ribonucleotide metabolic process | 5/48 | 401/18866 | 0.00342 | 0.019 | 0.012814 | 5 |
| BP | GO:0046660 | female sex differentiation | 3/48 | 119/18866 | 0.003442 | 0.019042 | 0.012842 | 3 |
| BP | GO:0033144 | negative regulation of intracellular steroid hormone receptor signaling pathway | 2/48 | 35/18866 | 0.003575 | 0.019462 | 0.013126 | 2 |
| BP | GO:0048333 | mesodermal cell differentiation | 2/48 | 35/18866 | 0.003575 | 0.019462 | 0.013126 | 2 |
| BP | GO:0098801 | regulation of renal system process | 2/48 | 35/18866 | 0.003575 | 0.019462 | 0.013126 | 2 |
| BP | GO:1900077 | negative regulation of cellular response to insulin stimulus | 2/48 | 35/18866 | 0.003575 | 0.019462 | 0.013126 | 2 |
| BP | GO:0009259 | ribonucleotide metabolic process | 5/48 | 416/18866 | 0.003998 | 0.02168 | 0.014622 | 5 |
| BP | GO:0006911 | phagocytosis, engulfment | 3/48 | 126/18866 | 0.004041 | 0.021824 | 0.014719 | 3 |
| BP | GO:0001558 | regulation of cell growth | 5/48 | 420/18866 | 0.004164 | 0.022398 | 0.015106 | 5 |
| BP | GO:1900026 | positive regulation of substrate adhesion-dependent cell spreading | 2/48 | 38/18866 | 0.004203 | 0.022523 | 0.01519 | 2 |
| BP | GO:0042180 | cellular ketone metabolic process | 4/48 | 260/18866 | 0.004263 | 0.02275 | 0.015343 | 4 |
| BP | GO:0016601 | Rac protein signal transduction | 2/48 | 39/18866 | 0.004423 | 0.023425 | 0.015798 | 2 |
| BP | GO:0071709 | membrane assembly | 2/48 | 39/18866 | 0.004423 | 0.023425 | 0.015798 | 2 |
| BP | GO:0019693 | ribose phosphate metabolic process | 5/48 | 427/18866 | 0.004465 | 0.023553 | 0.015885 | 5 |
| BP | GO:0045785 | positive regulation of cell adhesion | 5/48 | 428/18866 | 0.004509 | 0.023694 | 0.01598 | 5 |
| BP | GO:0032467 | positive regulation of cytokinesis | 2/48 | 40/18866 | 0.004649 | 0.024056 | 0.016224 | 2 |
| BP | GO:0045740 | positive regulation of DNA replication | 2/48 | 40/18866 | 0.004649 | 0.024056 | 0.016224 | 2 |
| BP | GO:0071526 | semaphorin-plexin signaling pathway | 2/48 | 40/18866 | 0.004649 | 0.024056 | 0.016224 | 2 |
| BP | GO:0072210 | metanephric nephron development | 2/48 | 40/18866 | 0.004649 | 0.024056 | 0.016224 | 2 |
| BP | GO:0007498 | mesoderm development | 3/48 | 134/18866 | 0.004799 | 0.02474 | 0.016686 | 3 |
| BP | GO:0032273 | positive regulation of protein polymerization | 3/48 | 137/18866 | 0.005104 | 0.026213 | 0.017679 | 3 |
| BP | GO:0045927 | positive regulation of growth | 4/48 | 274/18866 | 0.005129 | 0.026244 | 0.0177 | 4 |
| BP | GO:0006163 | purine nucleotide metabolic process | 5/48 | 442/18866 | 0.005162 | 0.026313 | 0.017746 | 5 |
| BP | GO:0006638 | neutral lipid metabolic process | 3/48 | 138/18866 | 0.005208 | 0.026351 | 0.017771 | 3 |
| BP | GO:0006639 | acylglycerol metabolic process | 3/48 | 138/18866 | 0.005208 | 0.026351 | 0.017771 | 3 |
| BP | GO:0034504 | protein localization to nucleus | 4/48 | 277/18866 | 0.005329 | 0.026863 | 0.018117 | 4 |
| BP | GO:0021762 | substantia nigra development | 2/48 | 43/18866 | 0.005356 | 0.026897 | 0.01814 | 2 |
| BP | GO:0021575 | hindbrain morphogenesis | 2/48 | 44/18866 | 0.005602 | 0.027824 | 0.018765 | 2 |
| BP | GO:0035307 | positive regulation of protein dephosphorylation | 2/48 | 44/18866 | 0.005602 | 0.027824 | 0.018765 | 2 |
| BP | GO:0044091 | membrane biogenesis | 2/48 | 44/18866 | 0.005602 | 0.027824 | 0.018765 | 2 |
| BP | GO:0007520 | myoblast fusion | 2/48 | 45/18866 | 0.005853 | 0.028966 | 0.019535 | 2 |
| BP | GO:0007043 | cell-cell junction assembly | 3/48 | 147/18866 | 0.006204 | 0.030592 | 0.020632 | 3 |
| BP | GO:0031503 | protein-containing complex localization | 4/48 | 291/18866 | 0.006332 | 0.031112 | 0.020983 | 4 |
| BP | GO:0006606 | protein import into nucleus | 3/48 | 150/18866 | 0.006559 | 0.032112 | 0.021657 | 3 |
| BP | GO:0072521 | purine-containing compound metabolic process | 5/48 | 472/18866 | 0.006783 | 0.03309 | 0.022317 | 5 |
| BP | GO:0007409 | axonogenesis | 5/48 | 482/18866 | 0.007396 | 0.035948 | 0.024244 | 5 |
| BP | GO:0046460 | neutral lipid biosynthetic process | 2/48 | 51/18866 | 0.007465 | 0.036028 | 0.024298 | 2 |
| BP | GO:0046463 | acylglycerol biosynthetic process | 2/48 | 51/18866 | 0.007465 | 0.036028 | 0.024298 | 2 |
| BP | GO:0030168 | platelet activation | 3/48 | 158/18866 | 0.007566 | 0.036385 | 0.024539 | 3 |
| BP | GO:0034660 | ncRNA metabolic process | 5/48 | 493/18866 | 0.008113 | 0.038878 | 0.02622 | 5 |
| BP | GO:0001954 | positive regulation of cell-matrix adhesion | 2/48 | 55/18866 | 0.008639 | 0.041253 | 0.027822 | 2 |
| BP | GO:0031295 | T cell costimulation | 2/48 | 56/18866 | 0.008944 | 0.042424 | 0.028612 | 2 |
| BP | GO:0060538 | skeletal muscle organ development | 3/48 | 168/18866 | 0.008946 | 0.042424 | 0.028612 | 3 |
| BP | GO:0006470 | protein dephosphorylation | 4/48 | 323/18866 | 0.009079 | 0.042905 | 0.028936 | 4 |
| BP | GO:0030307 | positive regulation of cell growth | 3/48 | 171/18866 | 0.009387 | 0.044055 | 0.029712 | 3 |
| BP | GO:0051170 | import into nucleus | 3/48 | 171/18866 | 0.009387 | 0.044055 | 0.029712 | 3 |
| BP | GO:0000768 | syncytium formation by plasma membrane fusion | 2/48 | 58/18866 | 0.009569 | 0.044149 | 0.029775 | 2 |
| BP | GO:0002011 | morphogenesis of an epithelial sheet | 2/48 | 58/18866 | 0.009569 | 0.044149 | 0.029775 | 2 |
| BP | GO:0031294 | lymphocyte costimulation | 2/48 | 58/18866 | 0.009569 | 0.044149 | 0.029775 | 2 |
| BP | GO:0035306 | positive regulation of dephosphorylation | 2/48 | 58/18866 | 0.009569 | 0.044149 | 0.029775 | 2 |
| BP | GO:0140253 | cell-cell fusion | 2/48 | 58/18866 | 0.009569 | 0.044149 | 0.029775 | 2 |
| BP | GO:0099173 | postsynapse organization | 3/48 | 173/18866 | 0.009688 | 0.044545 | 0.030043 | 3 |
| BP | GO:0043525 | positive regulation of neuron apoptotic process | 2/48 | 59/18866 | 0.009889 | 0.045165 | 0.030461 | 2 |
| BP | GO:0048008 | platelet-derived growth factor receptor signaling pathway | 2/48 | 59/18866 | 0.009889 | 0.045165 | 0.030461 | 2 |
| BP | GO:0006949 | syncytium formation | 2/48 | 60/18866 | 0.010214 | 0.046492 | 0.031355 | 2 |
| BP | GO:0048857 | neural nucleus development | 2/48 | 62/18866 | 0.010877 | 0.049345 | 0.03328 | 2 |
| BP | GO:0031122 | cytoplasmic microtubule organization | 2/48 | 63/18866 | 0.011215 | 0.050712 | 0.034201 | 2 |
| BP | GO:0050770 | regulation of axonogenesis | 3/48 | 186/18866 | 0.011782 | 0.053095 | 0.035809 | 3 |
| BP | GO:0006940 | regulation of smooth muscle contraction | 2/48 | 65/18866 | 0.011906 | 0.053336 | 0.035971 | 2 |
| BP | GO:0030336 | negative regulation of cell migration | 4/48 | 350/18866 | 0.011927 | 0.053336 | 0.035971 | 4 |
| BP | GO:0008217 | regulation of blood pressure | 3/48 | 187/18866 | 0.011953 | 0.053336 | 0.035971 | 3 |
| BP | GO:0046626 | regulation of insulin receptor signaling pathway | 2/48 | 66/18866 | 0.012259 | 0.054523 | 0.036772 | 2 |
| BP | GO:0043534 | blood vessel endothelial cell migration | 3/48 | 189/18866 | 0.012299 | 0.054524 | 0.036772 | 3 |
| BP | GO:2000146 | negative regulation of cell motility | 4/48 | 365/18866 | 0.013732 | 0.060681 | 0.040925 | 4 |
| BP | GO:0017038 | protein import | 3/48 | 200/18866 | 0.014307 | 0.062608 | 0.042225 | 3 |
| BP | GO:0030705 | cytoskeleton-dependent intracellular transport | 3/48 | 200/18866 | 0.014307 | 0.062608 | 0.042225 | 3 |
| BP | GO:1901888 | regulation of cell junction assembly | 3/48 | 200/18866 | 0.014307 | 0.062608 | 0.042225 | 3 |
| BP | GO:0000281 | mitotic cytokinesis | 2/48 | 72/18866 | 0.014467 | 0.063109 | 0.042562 | 2 |
| BP | GO:0060541 | respiratory system development | 3/48 | 204/18866 | 0.01508 | 0.065572 | 0.044224 | 3 |
| BP | GO:0001707 | mesoderm formation | 2/48 | 75/18866 | 0.015631 | 0.067111 | 0.045261 | 2 |
| BP | GO:0033143 | regulation of intracellular steroid hormone receptor signaling pathway | 2/48 | 75/18866 | 0.015631 | 0.067111 | 0.045261 | 2 |
| BP | GO:0099518 | vesicle cytoskeletal trafficking | 2/48 | 75/18866 | 0.015631 | 0.067111 | 0.045261 | 2 |
| BP | GO:1900076 | regulation of cellular response to insulin stimulus | 2/48 | 75/18866 | 0.015631 | 0.067111 | 0.045261 | 2 |
| BP | GO:0048332 | mesoderm morphogenesis | 2/48 | 77/18866 | 0.016429 | 0.070314 | 0.047421 | 2 |
| BP | GO:0014706 | striated muscle tissue development | 4/48 | 389/18866 | 0.016969 | 0.072398 | 0.048827 | 4 |
| BP | GO:0042692 | muscle cell differentiation | 4/48 | 390/18866 | 0.017113 | 0.072572 | 0.048944 | 4 |
| BP | GO:0050870 | positive regulation of T cell activation | 3/48 | 214/18866 | 0.017117 | 0.072572 | 0.048944 | 3 |
| CC | GO:0005925 | focal adhesion | 14/48 | 415/19559 | 7.54E-13 | 8.79E-11 | 5.71E-11 | 14 |
| CC | GO:0030055 | cell-substrate junction | 14/48 | 423/19559 | 9.77E-13 | 8.79E-11 | 5.71E-11 | 14 |
| CC | GO:0031252 | cell leading edge | 12/48 | 421/19559 | 2.93E-10 | 1.76E-08 | 1.14E-08 | 12 |
| CC | GO:0005911 | cell-cell junction | 11/48 | 493/19559 | 2.27E-08 | 1.02E-06 | 6.64E-07 | 11 |
| CC | GO:0031080 | nuclear pore outer ring | 3/48 | 10/19559 | 1.64E-06 | 5.92E-05 | 3.84E-05 | 3 |
| CC | GO:0001726 | ruffle | 6/48 | 179/19559 | 4.82E-06 | 0.000145 | 9.38E-05 | 6 |
| CC | GO:0030027 | lamellipodium | 6/48 | 201/19559 | 9.36E-06 | 0.000241 | 0.000156 | 6 |
| CC | GO:0018995 | host cellular component | 4/48 | 65/19559 | 1.94E-05 | 0.000387 | 0.000251 | 4 |
| CC | GO:0043657 | host cell | 4/48 | 65/19559 | 1.94E-05 | 0.000387 | 0.000251 | 4 |
| CC | GO:0032153 | cell division site | 4/48 | 72/19559 | 2.91E-05 | 0.000523 | 0.000339 | 4 |
| CC | GO:0005643 | nuclear pore | 4/48 | 85/19559 | 5.59E-05 | 0.000914 | 0.000593 | 4 |
| CC | GO:0031256 | leading edge membrane | 5/48 | 175/19559 | 6.79E-05 | 0.001019 | 0.000661 | 5 |
| CC | GO:0032587 | ruffle membrane | 4/48 | 95/19559 | 8.63E-05 | 0.001195 | 0.000775 | 4 |
| CC | GO:0005938 | cell cortex | 6/48 | 305/19559 | 9.68E-05 | 0.001245 | 0.000808 | 6 |
| CC | GO:0005903 | brush border | 4/48 | 106/19559 | 0.000132 | 0.001485 | 0.000964 | 4 |
| CC | GO:0030863 | cortical cytoskeleton | 4/48 | 106/19559 | 0.000132 | 0.001485 | 0.000964 | 4 |
| CC | GO:0031253 | cell projection membrane | 6/48 | 344/19559 | 0.000187 | 0.001975 | 0.001282 | 6 |
| CC | GO:0070938 | contractile ring | 2/48 | 10/19559 | 0.000262 | 0.002621 | 0.001701 | 2 |
| CC | GO:0032154 | cleavage furrow | 3/48 | 54/19559 | 0.000315 | 0.002985 | 0.001938 | 3 |
| CC | GO:0031314 | extrinsic component of mitochondrial inner membrane | 2/48 | 14/19559 | 0.000527 | 0.00474 | 0.003077 | 2 |
| CC | GO:0098862 | cluster of actin-based cell projections | 4/48 | 159/19559 | 0.000619 | 0.005309 | 0.003446 | 4 |
| CC | GO:0031965 | nuclear membrane | 5/48 | 301/19559 | 0.000832 | 0.006805 | 0.004418 | 5 |
| CC | GO:0043197 | dendritic spine | 4/48 | 175/19559 | 0.000886 | 0.00691 | 0.004486 | 4 |
| CC | GO:0044309 | neuron spine | 4/48 | 177/19559 | 0.000925 | 0.00691 | 0.004486 | 4 |
| CC | GO:0030139 | endocytic vesicle | 5/48 | 313/19559 | 0.000991 | 0.00691 | 0.004486 | 5 |
| CC | GO:0030864 | cortical actin cytoskeleton | 3/48 | 80/19559 | 0.000998 | 0.00691 | 0.004486 | 3 |
| CC | GO:0098858 | actin-based cell projection | 4/48 | 220/19559 | 0.002057 | 0.013714 | 0.008902 | 4 |
| CC | GO:0000777 | condensed chromosome kinetochore | 3/48 | 106/19559 | 0.002241 | 0.013913 | 0.009031 | 3 |
| CC | GO:0030175 | filopodium | 3/48 | 106/19559 | 0.002241 | 0.013913 | 0.009031 | 3 |
| CC | GO:0008305 | integrin complex | 2/48 | 31/19559 | 0.002621 | 0.015724 | 0.010207 | 2 |
| CC | GO:0005884 | actin filament | 3/48 | 118/19559 | 0.003038 | 0.017641 | 0.011451 | 3 |
| CC | GO:0000779 | condensed chromosome, centromeric region | 3/48 | 122/19559 | 0.003338 | 0.018774 | 0.012187 | 3 |
| CC | GO:0098636 | protein complex involved in cell adhesion | 2/48 | 36/19559 | 0.003523 | 0.019216 | 0.012473 | 2 |
| CC | GO:0045177 | apical part of cell | 5/48 | 433/19559 | 0.004069 | 0.021539 | 0.013982 | 5 |
| CC | GO:0000776 | kinetochore | 3/48 | 137/19559 | 0.004619 | 0.023756 | 0.01542 | 3 |
| CC | GO:0005635 | nuclear envelope | 5/48 | 473/19559 | 0.005895 | 0.029477 | 0.019134 | 5 |
| CC | GO:0031312 | extrinsic component of organelle membrane | 2/48 | 49/19559 | 0.006444 | 0.03135 | 0.02035 | 2 |
| CC | GO:0019898 | extrinsic component of membrane | 4/48 | 306/19559 | 0.006656 | 0.031527 | 0.020465 | 4 |
| CC | GO:0005912 | adherens junction | 3/48 | 166/19559 | 0.007851 | 0.036234 | 0.02352 | 3 |
| CC | GO:0016459 | myosin complex | 2/48 | 57/19559 | 0.008637 | 0.038868 | 0.02523 | 2 |
| CC | GO:0101003 | ficolin-1-rich granule membrane | 2/48 | 61/19559 | 0.009842 | 0.04321 | 0.028049 | 2 |
| CC | GO:0030496 | midbody | 3/48 | 182/19559 | 0.010084 | 0.043219 | 0.028054 | 3 |
| CC | GO:0016324 | apical plasma membrane | 4/48 | 361/19559 | 0.011727 | 0.046487 | 0.030176 | 4 |
| CC | GO:0098978 | glutamatergic synapse | 4/48 | 361/19559 | 0.011727 | 0.046487 | 0.030176 | 4 |
| CC | GO:0001725 | stress fiber | 2/48 | 68/19559 | 0.012119 | 0.046487 | 0.030176 | 2 |
| CC | GO:0097517 | contractile actin filament bundle | 2/48 | 68/19559 | 0.012119 | 0.046487 | 0.030176 | 2 |
| CC | GO:0000775 | chromosome, centromeric region | 3/48 | 196/19559 | 0.012313 | 0.046487 | 0.030176 | 3 |
| CC | GO:0005819 | spindle | 4/48 | 367/19559 | 0.012396 | 0.046487 | 0.030176 | 4 |
| CC | GO:0005905 | clathrin-coated pit | 2/48 | 71/19559 | 0.013159 | 0.048339 | 0.031378 | 2 |
| CC | GO:0031594 | neuromuscular junction | 2/48 | 76/19559 | 0.014975 | 0.052854 | 0.034309 | 2 |
| CC | GO:0032432 | actin filament bundle | 2/48 | 76/19559 | 0.014975 | 0.052854 | 0.034309 | 2 |
| CC | GO:0042641 | actomyosin | 2/48 | 79/19559 | 0.016114 | 0.055779 | 0.036207 | 2 |
| CC | GO:0000793 | condensed chromosome | 3/48 | 222/19559 | 0.01715 | 0.058246 | 0.037809 | 3 |
| MF | GO:0017056 | structural constituent of nuclear pore | 4/48 | 28/18352 | 8.05E-07 | 0.000129 | 0.000101 | 4 |
| MF | GO:0005178 | integrin binding | 6/48 | 144/18352 | 1.97E-06 | 0.000157 | 0.000123 | 6 |
| MF | GO:0003779 | actin binding | 7/48 | 437/18352 | 0.000131 | 0.006989 | 0.005472 | 7 |
| MF | GO:0031996 | thioesterase binding | 2/48 | 11/18352 | 0.000363 | 0.014517 | 0.011366 | 2 |
| MF | GO:0017016 | Ras GTPase binding | 6/48 | 415/18352 | 0.000709 | 0.020308 | 0.015899 | 6 |
| MF | GO:0031267 | small GTPase binding | 6/48 | 428/18352 | 0.000832 | 0.020308 | 0.015899 | 6 |
| MF | GO:0004659 | prenyltransferase activity | 2/48 | 17/18352 | 0.000888 | 0.020308 | 0.015899 | 2 |
| MF | GO:0030898 | actin-dependent ATPase activity | 2/48 | 24/18352 | 0.001782 | 0.035644 | 0.027905 | 2 |
| MF | GO:0051015 | actin filament binding | 4/48 | 206/18352 | 0.002039 | 0.03625 | 0.02838 | 4 |
| MF | GO:0000146 | microfilament motor activity | 2/48 | 29/18352 | 0.0026 | 0.041598 | 0.032567 | 2 |
| KEGG | hsa04510 | Focal adhesion | 44404 | 201/8108 | 3.02E-06 | 0.000236 | 0.000134 | 7 |
|  | hsa04810 | Regulation of actin cytoskeleton | 44404 | 218/8108 | 5.18E-06 | 0.000236 | 0.000134 | 7 |
|  | hsa04520 | Adherens junction | 44313 | 71/8108 | 8.14E-05 | 0.002468 | 0.001399 | 4 |
|  | hsa05100 | Bacterial invasion of epithelial cells | 44313 | 77/8108 | 0.000112 | 0.002544 | 0.001442 | 4 |
|  | hsa04530 | Tight junction | 44343 | 169/8108 | 0.000206 | 0.003756 | 0.002129 | 5 |
|  | hsa03013 | RNA transport | 44343 | 186/8108 | 0.000322 | 0.004889 | 0.002771 | 5 |
|  | hsa05130 | Pathogenic Escherichia coli infection | 44343 | 197/8108 | 0.00042 | 0.004933 | 0.002796 | 5 |
|  | hsa04670 | Leukocyte transendothelial migration | 44313 | 114/8108 | 0.000507 | 0.004933 | 0.002796 | 4 |
|  | hsa04015 | Rap1 signaling pathway | 44343 | 210/8108 | 0.000563 | 0.004933 | 0.002796 | 5 |
|  | hsa00130 | Ubiquinone and other terpenoid-quinone biosynthesis | 44254 | 11/8108 | 0.000577 | 0.004933 | 0.002796 | 2 |
|  | hsa04722 | Neurotrophin signaling pathway | 44313 | 119/8108 | 0.000596 | 0.004933 | 0.002796 | 4 |
|  | hsa05131 | Shigellosis | 44343 | 247/8108 | 0.001174 | 0.008903 | 0.005046 | 5 |
|  | hsa04360 | Axon guidance | 44313 | 182/8108 | 0.002878 | 0.019457 | 0.011028 | 4 |
|  | hsa04512 | ECM-receptor interaction | 44282 | 88/8108 | 0.002993 | 0.019457 | 0.011028 | 3 |
|  | hsa04933 | AGE-RAGE signaling pathway in diabetic complications | 44282 | 100/8108 | 0.004295 | 0.023621 | 0.013388 | 3 |
|  | hsa05203 | Viral carcinogenesis | 44313 | 204/8108 | 0.004336 | 0.023621 | 0.013388 | 4 |
|  | hsa05205 | Proteoglycans in cancer | 44313 | 205/8108 | 0.004413 | 0.023621 | 0.013388 | 4 |
|  | hsa04151 | PI3K-Akt signaling pathway | 44343 | 354/8108 | 0.005627 | 0.028446 | 0.016123 | 5 |
|  | hsa05014 | Amyotrophic lateral sclerosis | 44343 | 365/8108 | 0.006399 | 0.030222 | 0.01713 | 5 |
|  | hsa04014 | Ras signaling pathway | 44313 | 232/8108 | 0.006831 | 0.030222 | 0.01713 | 4 |
|  | hsa04071 | Sphingolipid signaling pathway | 44282 | 119/8108 | 0.006974 | 0.030222 | 0.01713 | 3 |
|  | hsa05135 | Yersinia infection | 44282 | 137/8108 | 0.010262 | 0.042447 | 0.024059 | 3 |
|  | hsa04370 | VEGF signaling pathway | 44254 | 59/8108 | 0.016259 | 0.064331 | 0.036463 | 2 |
|  | hsa05211 | Renal cell carcinoma | 44254 | 69/8108 | 0.021844 | 0.079737 | 0.045195 | 2 |
|  | hsa05120 | Epithelial cell signaling in Helicobacter pylori infection | 44254 | 70/8108 | 0.022441 | 0.079737 | 0.045195 | 2 |
|  | hsa05165 | Human papillomavirus infection | 44313 | 331/8108 | 0.022782 | 0.079737 | 0.045195 | 4 |
|  | hsa04062 | Chemokine signaling pathway | 44282 | 192/8108 | 0.025185 | 0.084113 | 0.047675 | 3 |
|  | hsa05212 | Pancreatic cancer | 44254 | 76/8108 | 0.026162 | 0.084113 | 0.047675 | 2 |
|  | hsa05412 | Arrhythmogenic right ventricular cardiomyopathy | 44254 | 77/8108 | 0.026805 | 0.084113 | 0.047675 | 2 |

**Table S3: Clinical information of four cohorts**

| clinical characteristics | | Training cohort  (n=259) | | Internal validation cohort  (n=259) | | | Total TCGA cohort  (n=518) | | External validation cohort  (n=39) | | |
| --- | --- | --- | --- | --- | --- | --- | --- | --- | --- | --- | --- |
| Age | ≤60 | 137 | 52.90% | 124 | 47.88% | 261 | | 50.39% | | 16 | 41.03% |
|  | >60 | 122 | 47.10% | 135 | 52.12% | 257 | | 49.61% | | 23 | 58.97% |
| Gender | female | 88 | 33.98% | 89 | 34.36% | 177 | | 34.17% | | NA | NA |
|  | male | 171 | 66.02% | 170 | 65.64% | 341 | | 65.83% | | NA | NA |
| Stage | I-II | 165 | 63.71% | 149 | 57.53% | 314 | | 60.62% | | NA | NA |
|  | III-IV | 94 | 36.29% | 110 | 42.47% | 204 | | 39.38% | | NA | NA |
| Grade | I-II | 120 | 46.33% | 120 | 46.33% | 240 | | 46.33% | | 13 | 33.33% |
|  | III-IV | 139 | 53.67% | 139 | 53.67% | 278 | | 53.67% | | 26 | 66.67% |
| T | T1-2 | 175 | 67.57% | 157 | 60.62% | 332 | | 64.09% | | 16 | 41.03% |
|  | T3-4 | 84 | 32.43% | 102 | 39.38% | 186 | | 35.91% | | 23 | 58.97% |
| M | M0 | 208 | 80.31% | 206 | 79.54% | 414 | | 79.92% | | 25 | 64.10% |
|  | M1-X | 51 | 19.69% | 53 | 20.46% | 104 | | 20.08% | | 14 | 35.90% |
| N | N0 | 122 | 47.10% | 113 | 43.63% | 235 | | 45.37% | | 31 | 79.49% |
|  | N1-X | 137 | 52.90% | 146 | 56.37% | 283 | | 54.63% | | 8 | 20.51% |
| Total |  | 259 | 100.00% | 259 | 100.00% | 518 | | 100.00% | | 39 | 100.00% |


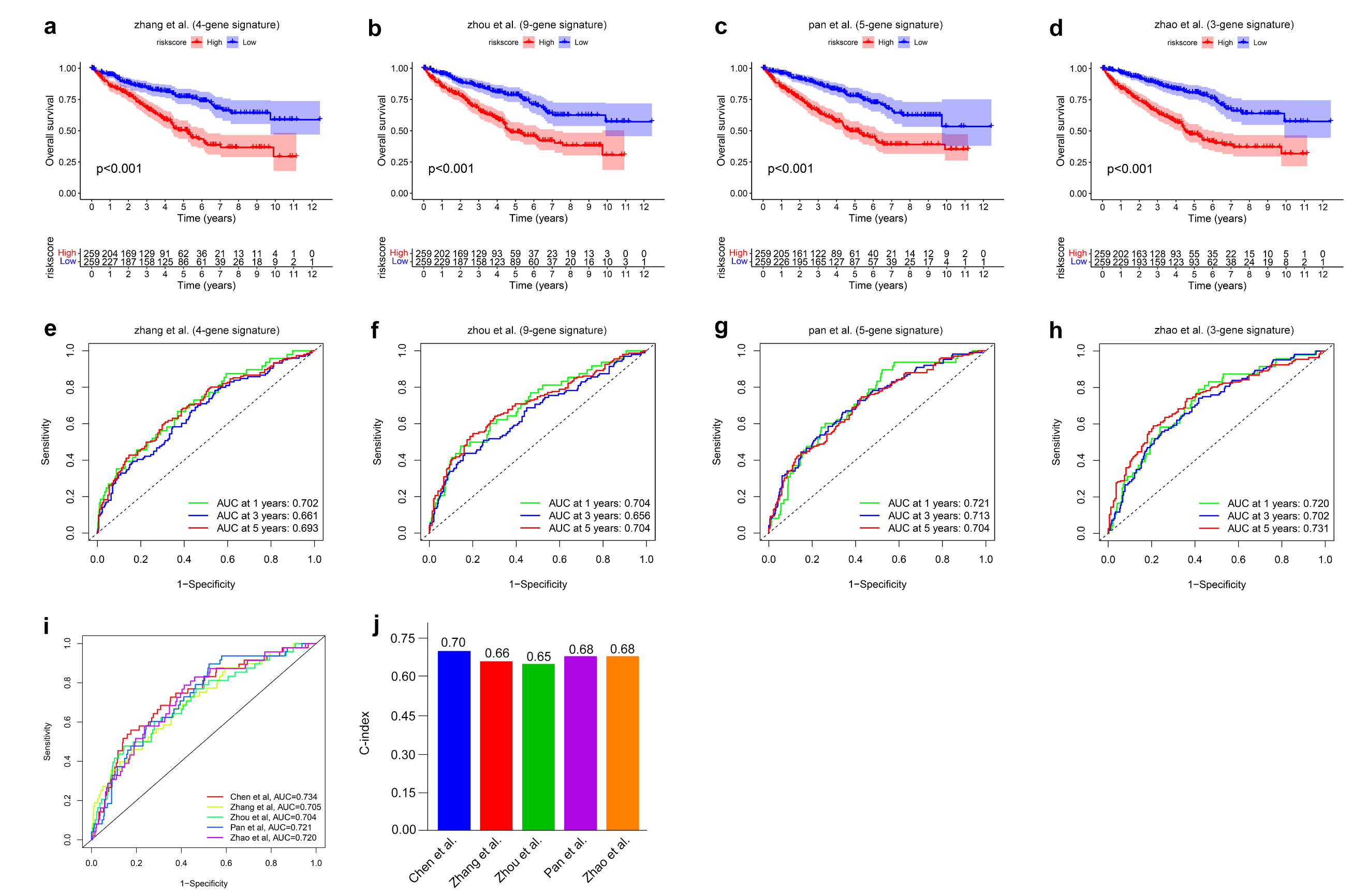


**Figure S1.** Comparison of our model with other four KIRC models. **a-d.** Kaplan-Meier survival analysis of OS in high-risk (red) and low-risk(blue) patients in a four-gene signature(**a**), a nine-gene signature (**b**), a five-gene signature (**c**) and a three-gene signature (**d**). **e-h.** ROC curves for predicting 1-, 3-, 5-year OS in a four-gene signature (**e**), a nine-gene signature (**f**), a five-gene signature (**g**) and a three-gene signature (**h**). **i**. ROC curves of the five prognostic risk models including our model. **j**. C-index of the five prognostic risk models including our model.


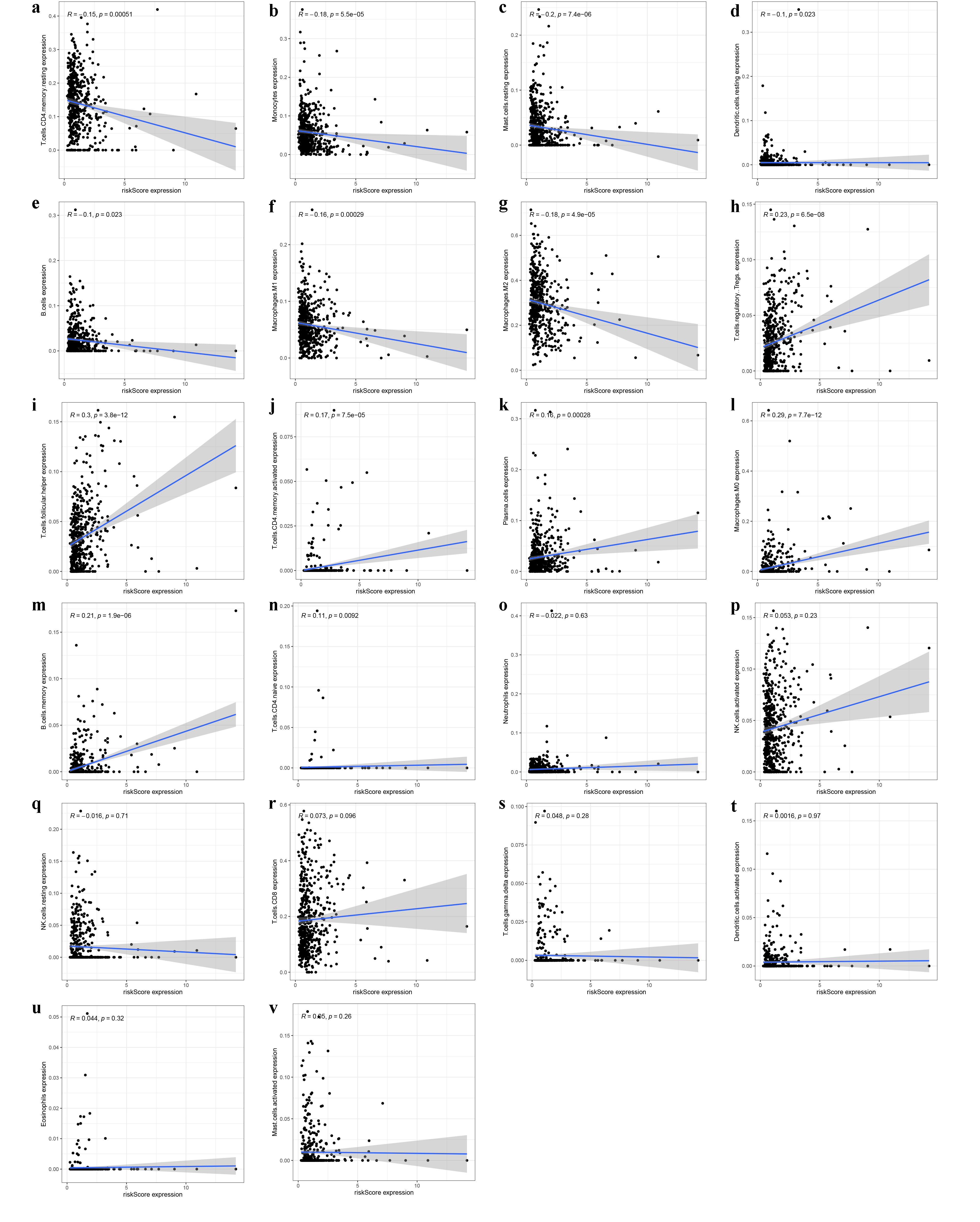


**Figure S2.** Relationships of risk score with 22 immune cells, including resting memory CD4+ T cells (**a**), monocytes (**b**), resting mast cells (**c**), resting dendritic cells (**d**), B cells (**e**), macrophages M1 (**f**), macrophages M2 (**g**), regulatory T cells (**h**), follicular helper T cells (**i**), activated memory CD4+ T cells (**j**), plasma cells (**k**), macrophages M0 (**l**), memory B cells (**m**), naive CD4+ T cells (**n**), neutrophils (**o**), activated NK cells (**p**), resting NK cells (**q**), CD8+ T cells (**r**), gamma delta T cells (**s**), activated memory CD4+T cells (**t**), eosinophils(**u**), activated mast cells (**v**).


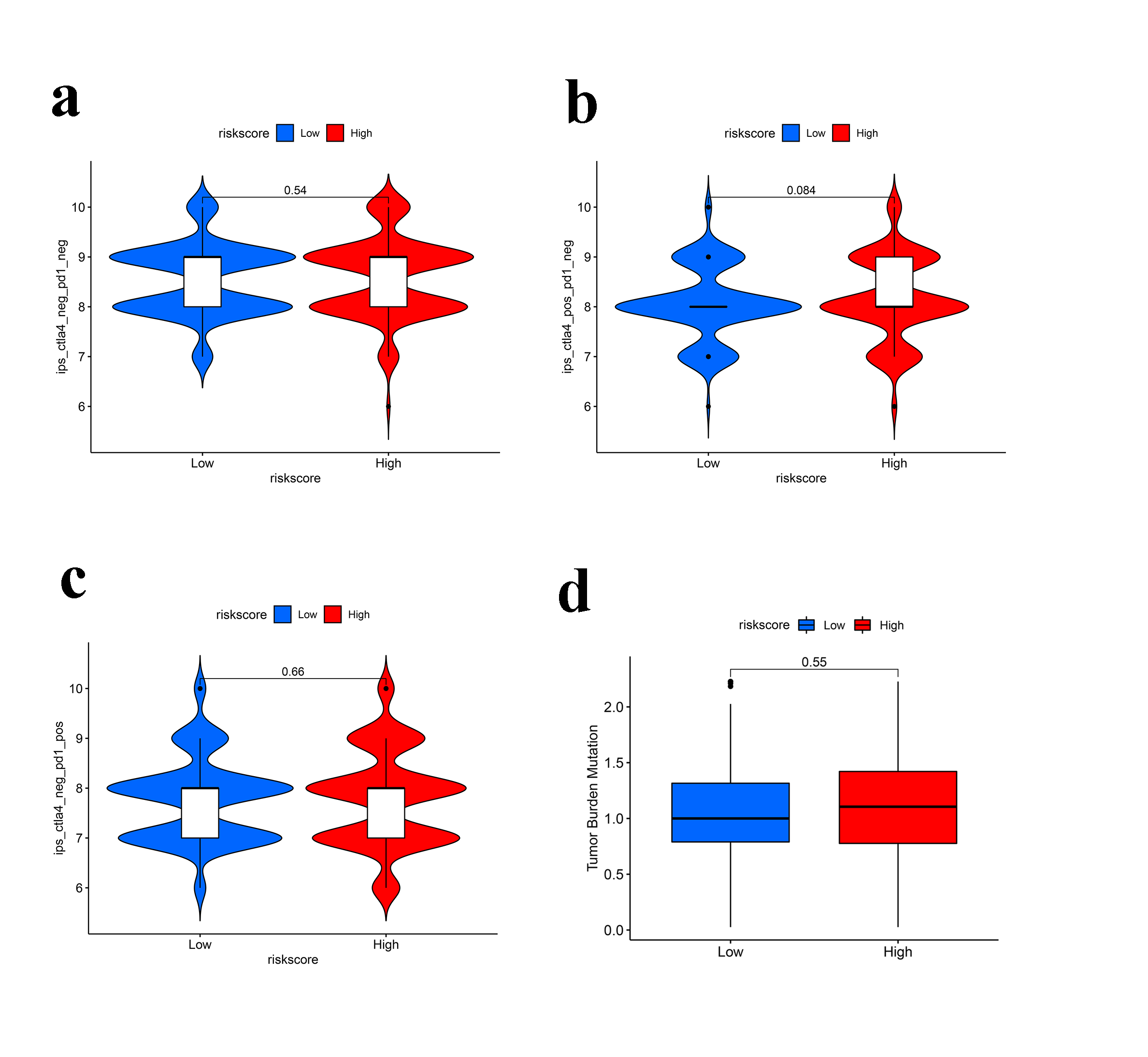


**Figure S3.** THE IPS and TMB among low-risk and high-risk groups. **a-c** The association between IPS and risk score in KIRC patients, including ips_ctla4_neg_pd1_neg (**a**), ips_ctla4_pos_pd1_neg (**b**)，ips_ctla4_neg_pd1_pos (**c**). **d**. The relationship between risk score and TMB.
